# Various mutations compensate for a deleterious lacZα insert in the replication enhancer of M13 bacteriophage

**DOI:** 10.1371/journal.pone.0176421

**Published:** 2017-04-26

**Authors:** Emily M. Zygiel, Karen A. Noren, Marta A. Adamkiewicz, Richard J. Aprile, Heather K. Bowditch, Christine L. Carroll, Maria Abigail S. Cerezo, Adelle M. Dagher, Courtney R. Hebert, Lauren E. Hebert, Gloria M. Mahame, Stephanie C. Milne, Kelly M. Silvestri, Sara E. Sutherland, Alexandria M. Sylvia, Caitlyn N. Taveira, David J. VanValkenburgh, Christopher J. Noren, Marilena Fitzsimons Hall

**Affiliations:** 1 Department of Chemistry, Stonehill College, Easton, Massachusetts, United States of America; 2 New England Biolabs, Inc., Ipswich, Massachusetts, United States of America; US Naval Research Laboratory, UNITED STATES

## Abstract

M13 and other members of the Ff class of filamentous bacteriophages have been extensively employed in myriad applications. The Ph.D. series of phage-displayed peptide libraries were constructed from the M13-based vector M13KE. As a direct descendent of M13mp19, M13KE contains the lacZα insert in the intergenic region between genes IV and II, where it interrupts the replication enhancer of the (+) strand origin. Phage carrying this 816-nucleotide insert are viable, but propagate in *E*. *coli* at a reduced rate compared to wild-type M13 phage, presumably due to a replication defect caused by the insert. We have previously reported thirteen compensatory mutations in the 5’-untranslated region of gene II, which encodes the replication initiator protein gIIp. Here we report several additional mutations in M13KE that restore a wild-type propagation rate. Several clones from constrained-loop variable peptide libraries were found to have ejected the majority of lacZα gene in order to reconstruct the replication enhancer, albeit with a small scar. In addition, new point mutations in the gene II 5’-untranslated region or the gene IV coding sequence have been spontaneously observed or synthetically engineered. Through phage propagation assays, we demonstrate that all these genetic modifications compensate for the replication defect in M13KE and restore the wild-type propagation rate. We discuss the mechanisms by which the insertion and ejection of the lacZα gene, as well as the mutations in the regulatory region of gene II, influence the efficiency of replication initiation at the (+) strand origin. We also examine the presence and relevance of fast-propagating mutants in phage-displayed peptide libraries.

## Introduction

Recent publications underscore considerable and ongoing interest in filamentous bacteriophages. Their distinctive properties (*e*.*g*., a robust non-lytic life cycle, accommodation of genomic inserts via a flexible capsid length, solvent-accessible coat protein N-termini amenable to protein display) continually yield new and myriad practical applications in the areas of drug delivery, disease diagnostics, treatment of cancer and neurodegenerative diseases, and synthesis of biological, chemical, and nano materials (as described in these reviews, articles, and references therein [1–6]). While decades of research have revealed a seemingly comprehensive body of information about these phages, investigation into their fundamental biology actively continues in such areas as phage structure and assembly [7,8], control mechanisms in the viral life cycle [9], and impact on bacterial hosts [10–12]. As relatively simple model systems, the filamentous bacteriophages can be employed to elucidate key cellular processes such as DNA replication, transcription, and translation, providing insights that can be extended to more complex organisms.

**Fig 1:**
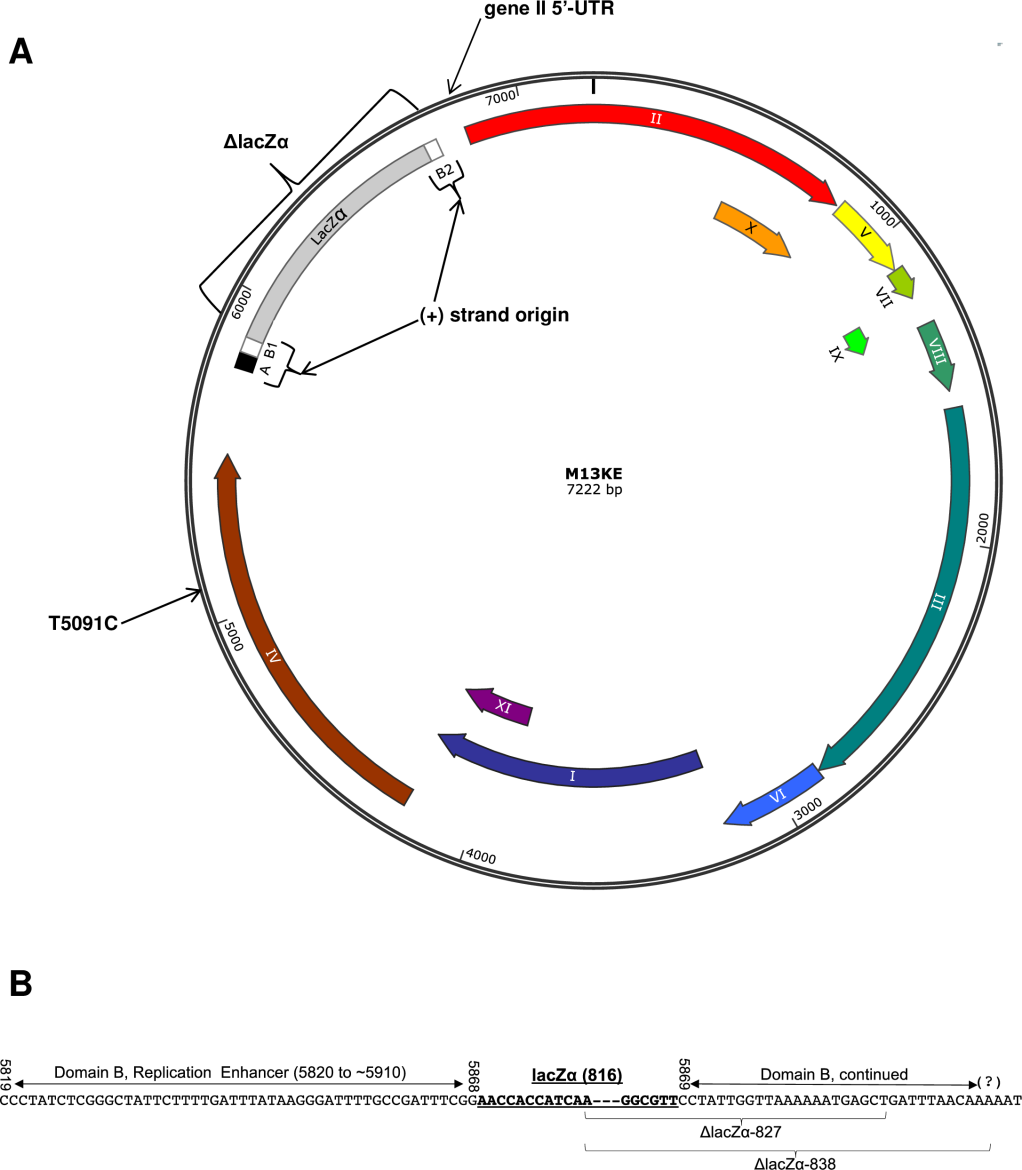
Schematics of the M13KE genome. (A) The map of M13KE is shown. The (+) strand origin is divided into Domains A and B [20]. Domain A (nucleotides 5769–5819 in both WT-M13 and M13KE) is the “core origin” and is required for both (+) strand initiation and termination. Domain A is extremely sensitive to deletions and insertions, which reduce biological activity to ≤ 0.01% [20]. Domain B stretches from position 5820 to about 5910 in wild-type M13 (WT-M13), but it is interrupted in M13KE by the lacZα insert (the separated segments are indicated as B1 and B2). Dubbed the “replication enhancer,” Domain B is required for (+) strand initiation and is moderately sensitive to inserts and deletions, which reduce biological activity to ≥ 1% [20,31]. The locations of spontaneous mutations and ejections in M13KE are labeled as gene II 5’-UTR, ΔlacZα, and T5091C. The T5091C mutation is a reversion back to the WT-M13 nucleotide at position 5092 (the number is lower by 1 nt due a missing 1565T in M13mp18,19 and M13KE). The map of WT-M13 would be identical to M13KE with the exception of the lacZα insert (all downstream numbering is 815 nt lower in WT-M13). The (-) strand origin (not labeled) is upstream of the (+) strand origin in the intergenic region. The map was constructed using SnapGene^®^. (B) The exact locations of the lacZα insert and ejections are indicated. Nucleotide numbering corresponds to WT-M13. Domain A is not shown except for last downstream base, 5819C. In M13mp-based phage, an 816-nt insert containing the lacZα gene is placed in Domain B between nucleotides 5868 and 5869. Two different spontaneous ejections have arisen in M13KE, ΔlacZα-827 and ΔlacZα-838, both of which left behind eleven nucleotides of the lacZα insert. The vast majority of the lacZα insert was removed, in addition to a small section of Domain B: 22 nucleotides in the smaller (827-nt) ejection and 33 nt in the larger (838-nt) ejection.

The members of the Ff class of filamentous bacteriophages (M13, fd, and f1) possess 98.5% genomic identity, produce nearly identical proteins, and are characterized by the same life-cycle processes, including the replication of the viral genome [13,14]. Following infection of the *Escherichia coli* (*E*. *coli*) host cell, the single-stranded genome (the (+) strand) is converted into the double-stranded replicative form (RF I) [15,16]. Replication of the (+) strand is initiated in the intergenic region (IG) between genes IV and II, located at positions 5498–6005 in the phage genome ([17], and see note in [18]). The initiator protein, gIIp (gene II protein) binds to RF I with a footprint that covers nucleotides 5774–5813 in Domain A of the (+) strand origin (Fig 1A) [19,20]. The gIIp recognition sequence spans from position 5777 [21] to somewhere in the range of 5791–5809 [20]. Then gIIp nicks the (+) strand between 5780T and 5781A [22,23] and, simultaneously, Tyr-197 is covalently linked to the 5’-end of the nicked strand [24]. In addition, gIIp forms complex with *E*. *coli* rep helicase and DNA binding protein I to assist in the unwinding of the double-stranded DNA at the replication fork [25]. DNA polymerase III extends the 3’-end of the (+) strand using the (-) strand as a template, and thereby rolls off the (+) strand from the gIIp-ligated 5’-end [15]. Following one round of replication, the gIIp-bound 5’-end is transferred from the enzyme to the liberated 3’-end of the same strand to produce a closed single (+) strand [26]. In the early stages of the infection, the newly synthesized (+) strand is converted to RF I to repeat the replication cycle, but once gene V protein (gVp) reaches a threshold concentration, it coats the single strand in preparation for packaging [14,27,28]. To further control DNA replication, gVp also represses the production of gIIp by binding to the gene II mRNA at the operator sequence, which consequently slows down replication and shifts the life cycle to the assembly of progeny phage particles [29,30].

The highly efficient arrangement of the eleven genes in each of the Ff phage genomes leaves very little non-coding space. As a result, when these phage have been employed as recombinant DNA vectors, the inserts have generally been confined to the intergenic region in which the origin of replication (*ori*) is located [32]. The most widely used vectors have been the M13mp vectors constructed by Messing and coworkers, which have served as indispensible tools in the preparation, cloning, and sequencing of single-stranded DNA (for reviews, see [33,34]). The polylinker embedded in the gene for the α fragment of β-galactosidase (lacZα) can host various DNA fragments, each of which can be sequenced from a common primer annealed to the single-stranded genome isolated from the M13 phage virion. Advantageously, blue-white screening can be used to select clones with an insert that interrupts the lacZα gene. Messing’s original construct was M13mp1 [35], in which the lacZα gene was placed between positions 5868 and 5869 in Domain B of the *ori* (Fig 1A and 1B). The next step was to incorporate a useful restriction site in the lacZα gene; chemical mutagenesis afforded an EcoRI recognition sequence in M13mp2 [36]. Subsequent iterations led to the development of the full polylinker, comprised of ten unique restriction sites embedded in the lacZα gene in M13mp18 [37]. In addition to the polylinker-harboring lacZα insert, there are nineteen differences between wild-type M13 (WT-M13) and M13mp18 scattered throughout the genome (S1 Table), which we presume to be a mixture of spontaneous and intentionally engineered mutations incorporated during the development of the M13mp vectors.

M13mp19 (identical to M13mp18 with the exception of the reversed polylinker [37]) was modified to house the Ph.D. phage-displayed peptide libraries, in which randomized peptides are appended to the N-terminus of the coat protein gIIIp [38]. First, the small section of the polylinker between the HincII and SacII sites was deleted to remove the KpnI site. Subsequently, two rounds of Kunkel mutagenesis [39] were used to introduce KpnI and EagI cloning sites to gene III in the new vector, M13KE. In the Ph.D. libraries, displayed peptides are fused the N-terminus of gIIIp by inserting a randomized oligonucleotide between the KpnI and EagI cloning sites, which flank the junction between the coding sequences for the signal peptide and the mature protein [38].

We have previously reported spontaneously occurring clones from the Ph.D. libraries that propagate in *E*. *coli* cells significantly faster than M13KE phage as well as the pool of library phage from which they come [40,41]. The vast majority of these phage clones have single mutations or deletions in the 5’-untranslated region (5’-UTR) of gene II. Here we present several new mutations in the gene II 5’-UTR as well as one mutation in gene IV. We also report an alternative event in which M13KE ejects a portion of the lacZα insert from its genome. Each of these different genetic changes confers a fast-propagation phenotype to M13KE, with a rate equivalent to that of WT-M13. The lacZα ejections and 5’-UTR mutations have presumed relevance to the initiation of replication based on their respective connections to the (+) strand origin and the initiator protein gIIp. Compensatory mechanisms by which these genetic changes restore wild-type propagation rates are discussed.

## Materials and methods

### Materials

The Ph.D.-7 Phage Display Peptide Library (lot 3), *E*. *coli* ER2738 (*F´ proA*^*+*^*B*^*+*^ *lacI*^*q*^ *Δ(lacZ)M15 zzf*::*Tn10(Tet*^*R*^*)/fhuA2 glnV Δ(lac-proAB) thi-1 Δ(hsdS-mcrB)5*), M13KE phage, the M13KE gIII cloning vector, Phusion^®^ High-Fidelity DNA Polymerase, Q5^TM^ High-Fidelity Polymerase, Taq DNA Polymerase, T4 Polynucleotide Kinase, T4 DNA Ligase, restriction enzymes, the –96 gIII sequencing primer, and the primer with the randomized gene II operator sequence were supplied by New England Biolabs, Inc. (NEB, Ipswich, MA). All other custom primers for PCR and sequencing were synthesized by Integrated DNA Technologies, Inc. (Coralville, IA). *E*. *coli* bacteriophage M13 (WT-M13) was purchased from ATCC (Manassas, VA). X-gal (5-bromo-4-chloro-3-indolyl-β-D-galactopyranoside) and IPTG (isopropyl-β-D-thiogalactopyranoside) were from AmericanBio, Inc. (Natick, MA). Polyethylene glycol 8000 (PEG) was from Sigma-Aldrich, Inc. (St. Louis, MO). The QIAquick PCR Purification and Gel Extraction Kits and the QIAprep Spin Miniprep Kit were purchased from QIAGEN Inc. (Valencia, CA). The Miller formulation of lysogeny broth (LB) and all other materials and reagents were from Thermo Fisher Scientific (Waltham, MA).

### General methods

All methodology for the use of the Ph.D. libraries, including preparation of media and solutions, ER2738 strain maintenance, phage amplification, titering, and purification of single-stranded M13 viral DNA is described in the Ph.D.^TM^ Phage Display Libraries Instruction Manual [42] and in the literature [43]. Sanger dideoxy sequencing of DNA was performed by the New England Biolabs Sequencing Core Facility with an Applied Biosystems 3730xl DNA Analyzer, using a BigDye Terminator v.3.1 Cycle Sequencing Kit (Applied Biosystems, Foster City, CA). The randomized peptide region (fused to gene III) of Ph.D. library clones was sequenced using either the –96 gIII sequencing primer NEB1259 = 5’-d(CCCTCATAGTTAGCGTAACG) or the custom primer NOR751 = 5’-d(CCGTAACACTGAGTTTCGTCACC). The 5’-UTR of gene II was sequenced using primer NOR631 = 5’-d(GGCCGGAGACAGTCAAATCACC) and the region around position 5091 in M13KE was sequenced using the primer NOR5091 = 5’-d(CTTTGATTAGTAATAACATCACTTGCC). The double-stranded replicative-form (RF I) DNA of select phage clones (WT-M13, ΔlacZα-827, ΔlacZα-838, Ph-SRITIDN (G6793T), Ph-SPTQPKS (G6792C+T5091C), Ph-HAFPHLH (G6793Δ+T5091C), Ph-HAFPHLH (T5091C), Ph-NoPeptide (G6793Δ), T6797C+T6789Δ, and WT-C5092T) was purified from ER2738 cultures using the QIAprep Spin Miniprep Kit, and was sequenced using a panel of 20–36 custom primers. All statistical computations were carried out using JMP® Pro 12.0.1.

### Discovery of lacZα ejections in M13KE-based phage

Six disulfide-constrained, pIII-displayed, peptide-loop libraries, ACCX_6_, ACX_2_CX_4_, ACX_3_CX_3_, ACX_4_CX_2_, ACX_5_CX_1_, and ACX_6_C were constructed (K.A. Noren, unpublished) using a previously described method [38]. For each library, a targetless amplification procedure was used to screen for the presence of contaminating wild-type phage and other phage species having a propagation advantage. Briefly, phage (~10^6^ pfu = plaque-forming units) were added to 20 mL of a 100-fold diluted overnight culture of ER2738 in LB medium, and incubated for 4.5 h at 37°C with shaking (250 rpm). Infected culture (1 mL) was centrifuged twice at 18,000 g for 1 min, and the supernatant was titered to determine phage concentration. This procedure was repeated using ~10^6^ pfu produced by the first amplification. Following titering of the second round, phage DNA from white plaques was sequenced using primer NEB1259 to identify the peptide displayed on gIIIp. Primer NOR631 was used to sequence through the gene II 5’-UTR and upstream of it to determine the presence or absence of the lacZα insert. A mixture prepared by pooling the six loop libraries, along with the commercially available Ph.D.-C7C library (NEB), was used to pan against streptavidin as described previously [43]. Following three rounds of panning, phage DNA from white plaques was sequenced as described above.

### Identification of fast-propagating clones in a phage-displayed peptide library

Fast-propagating clones from the Ph.D.-7 library were identified as previously described [41]. Briefly, the Ph.D.-7 library was serially amplified three times. To screen for fast-propagating clones, twelve or more plaques from the third round of amplification were each used to infect 1 mL of early log ER2738 culture. After 135 minutes of shaking at 250 rpm and 37°C, 10^5^-fold and 10^6^-fold dilutions of each growing culture were plated and the plaques were counted the next day. The plaques from clones with particularly high numbers were amplified to purify the viral DNA, and the clones were identified by sequencing both the gene II 5’-UTR (NOR631) and the displayed peptide fused to gene III (NOR751).

### Fragment swap to isolate the T5091C mutation in M13KE

To isolate the T5091C mutation from the double mutant Ph-HAFPHLH, which contains both the T5091C mutation and a G6793Δ deletion [41], the Acc65I-to-EcoRI fragment of Ph-HAFPHLH (containing the T5091C mutation and the displayed peptide HAFPHLH) was ligated to the EcoRI-to-Acc65I fragment of M13KE (containing a G at position 6793). The Acc65I-to-EcoRI fragment was obtained by PCR using the single-stranded Ph-HAFPHLH genome (purified from a single plaque) as the template and the primers 5’-d(GCCTTTTTTTTGGAGATTTTCAACG) and 5’-d(GTCACGACGTTGTAAAACGACG), which annealed upstream of the EcoRI site (on the coding strand) and downstream of the Acc65I site (on the noncoding strand), respectively. PCR was performed using Phusion High-Fidelity DNA Polymerase with an annealing gradient of 45–70°C. The 4737-bp PCR product was purified using the QIAquick PCR Purification Kit, digested with EcoRI and BamHI, and finally purified from a 1% agarose gel using the QIAquick Gel Extraction Kit to give the 4647-bp fragment. The EcoRI-to-Acc65I fragment of M13KE was obtained by digesting the M13KE cloning vector with EcoRI and Acc65I, and purifying the 2575-bp fragment from a 1% agarose gel using the QIAquick Gel Extraction Kit. The two fragments were ligated at various ratios of Ph-HAFPHLH:M13KE (2:1 to 20:1) using T4 DNA Ligase at 16°C for 2 hours and then 16 hours (O/N) at room temperature. The ligation product (2–5 ng DNA) was used to transfect ER2738 cells, and the outgrowth was plated on LB/agar/IPTG/X-gal plates. Twelve plaques were amplified, and the purified single-stranded viral DNA was sequenced at the locations of the 5’-UTR of gene II (NOR631), the displayed peptide fused to gene III (NOR751), and position 5091 (NOR5091) to identify the desired clone.

### Incorporation of novel mutations into the gene 5’-UTR of M13KE

Mutations predicted to confer fast propagation rates were incorporated into the 5’-UTR of gene II by placing a mutagenized insert between the SwaI and BglII restriction sites in M13KE. The double-stranded M13KE cloning vector was digested with SwaI and BglII, and the products were electrophoresed on a 1% agarose gel, from which the 7071 bp DNA band was purified using the QIAquick Gel Extraction Kit.

The insert was built through polymerase cycling assembly (PCA) [44,45] using six overlapping primers named A through F, of which primer B contained the variation in the sequence in the gene II 5’-UTR. To incorporate the T6797C mutation, primer B was 5’-d(GTACCCCGGTTGATAATCAGAGAAGCCCCAAAAACAGGAAGATTGTATAAGC), in which the underlined G is mutated from A in the anticoding strand. To make the insert containing a randomized stretch of the gene II operator sequence, primer B was 5’-d(GTACCCCGGTTGATAATCAGAAAAGCCCCAAAAACAGGAAGATTGTATAAGC), in which each underlined position was a mixture of 88% of the correct DNA base with 4% of each incorrect base. In all cases, the upstream end primer A included half of the SwaI recognition sequence (shown in italics) in 5’-d(-*AAAT*ATTTGCTTATACAATCTTCCTG) to match the blunt end that resulted from SwaI digestion of the vector. The downstream end primer F included the complete BglII sequence (show in italics) preceded by a short extension in 5’-d(GCCTGCG*AGATCT*ACAAAGGCTATCAGGTCATTGCC) to allow digestion of this end of the insert by BamHI. The remaining primers were C = 5’-d(GATTATCAACCGGGGTACATATGATTGACATGCTAGTTTTACGATTACCGTTC), D = 5’-d(TCTGGAGCAAACAAGAGAATCGATGAACGGTAATCGTAAAAC), and E = 5’-d(TCTCTTGTTTGCTCCAGACTCTCAGGCAATGACCTGATAGCC). A 25 μL PCA reaction mixture contained 80 nM each of primers A through F, 0.2 mM dNTPs, and 0.625 units of Taq DNA Polymerase in 1X ThermoPol buffer. A thermocycler was used to subject the samples to 40 rounds of (i) denaturation at 95°C for 30 s, (ii) annealing with a 30–55°C gradient for 30 s, and (iii) extension at 68°C for 15 s. Electrophoresis in 1.8% agarose confirmed the presence of the 161 bp DNA duplex. The PCA product (0.1 μL) was amplified by a standard polymerase chain reaction (PCR) using just the end primers A and F and Q5 High-Fidelity Polymerase, with an annealing temperature gradient of 30–55°C. The PCR product was purified by phenol/chloroform extraction and ethanol precipitation. Digestion with BglII yielded the 150 bp insert, which was purified using the QIAquick Gel Extraction Kit.

Ligations were performed on a scale of 50 ng vector with various ratios of insert:vector (1, 3, 10, and 30:1). Samples were incubated with T4 DNA Ligase at 16°C for 5 hours and then 16 hours (O/N) at room temperature. ER2738 cells were transfected with the ligation mixture (~2.5 ng DNA), and the outgrowth was plated on LB/agar/IPTG/X-gal plates. Approximately twelve plaques were amplified, and the purified single-stranded viral DNA was sequenced at the location of the 5’-UTR of gene II to identify mutations.

### Incorporation of single mutations into wild-type M13

Each mutation was incorporated into wild-type M13 by PCR of the entire genome with the desired mutation contained in one of the primers. The template was the single-stranded WT-M13 genome as purified from a single plaque. The two PCR primers were positioned back-to-back (5’-end to 5’-end) in order to synthesize a double-stranded version of the entire genome. For the G6813A mutation, the primers were 5’-d(AGAAAAGCCCCAAAAACAGGAAGATTG) (counterclockwise) and 5’-d(GATTATCAACCGAGGTACATATGATTG) (clockwise), where the underlined A in the latter primer provided the G6813A mutation. The A6802T mutation employed the same counterclockwise primer, but the clockwise primer containing the mutation was 5’-d(GTTTATCAACCGGGGTACATATGATTG). For the G6792T mutation, the mutation was incorporated into the counterclockwise primer 5’-d(AGAAAAGCACCAAAAACAGGAAGATTG), while the clockwise primer 5’-d(GATTATCAACCGGGGTACATATGATTG) contained the normal sequence. The C5092T mutant employed the primers 5’-d(CAGAAGGGTTCTATCTCTGTTGG) (forward primer) and 5’-d(CCAGTAATAAAAGGAACATTCTGG) (reverse primer containing mutation). PCR was performed using Phusion High-Fidelity DNA Polymerase with an annealing gradient of 48–72°C. The 6407 bp PCR product was electrophoresed on a 1% agarose gel, from which it was purified using a QIAquick Gel Extraction Kit. The purified PCR product (100–200 ng) was incubated with T4 Polynucleotide Kinase at 37°C for 30–60 minutes to phosphorylate the 5’ ends, followed by circularization with T4 DNA Ligase for 4–6 hours at 16°C and 16 hours (O/N) at room temperature. Ligation mixture containing approximately 5 ng DNA was used to transfect ER2738 cells, and the outgrowth was plated on LB/agar/IPTG/X-gal plates. Approximately ten plaques were amplified, and the purified single-stranded viral DNA was sequenced at the location of the 5’-UTR of gene II to confirm the presence of the mutation.

### Comparison of propagation rates

Before each propagation experiment, a concentrated stock solution of each phage clone was prepared by amplifying approximately 1 x 10^8^ pfu of the particular phage in 20 mL of early log ER2738 cells. The solution was titered by standard methods [42,43] to determine phage concentration from plaque counts. All clone identities were carefully verified by purifying and sequencing the viral DNA from plaques. In the propagation experiment, 1 x 10^8^ virions of each clone were combined with 20 mL of early log ER2738 culture and shaken at 250 rpm and 37°C. At various time points, 10 μL of an appropriate dilution of the culture was plated, and plaques were counted the following day.

## Results

### Effect of the insertion and ejection of the lacZα gene on M13-based phage

**Fig 2:**
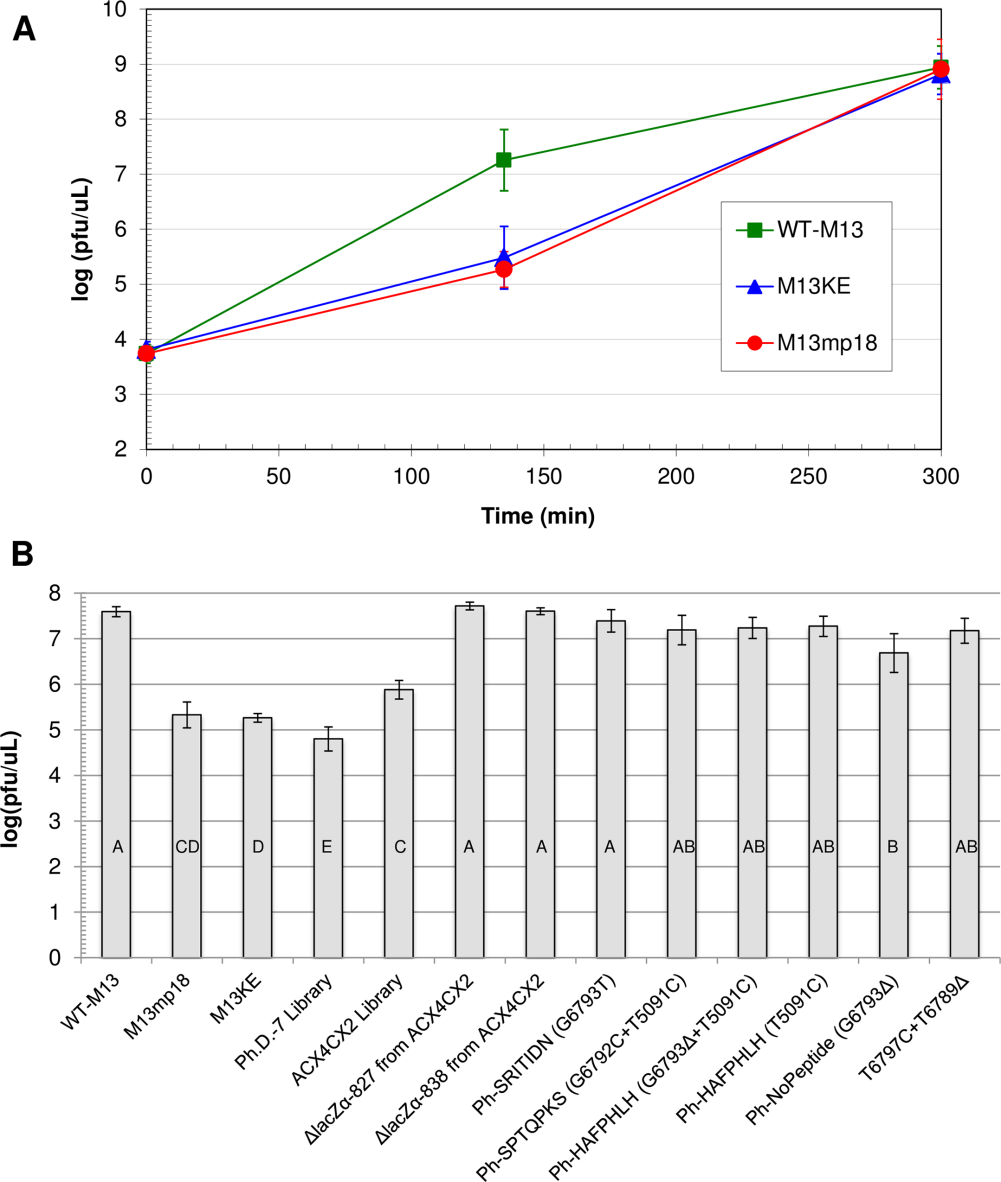
Comparison of propagation rates for various M13-based phage. (A) Time course for amplification of WT-M13, M13KE, and M13mp18. Each phage clone was amplified in three separate early log cultures of *E*. *coli* ER2738. From each culture, one aliquot was diluted and plated at the indicated incubation times, and the concentration of phage (pfu/μL) in each growing culture was determined based on plaque counts. Data points represent the mean log(pfu/μL) of the three separate cultures for each type of phage, and the error bars show the 95% confidence interval. Statistical analysis indicated significant differences among the phage concentrations for the three sets of data at 135 minutes (ANOVA; F_**2,8**_ = 89.2, P < 0.0001). Post-hoc analysis showed that M13KE and M13mp18 are not significantly different from each other (Tukey’s HSD; α = 0.05, P = 0.4471). Both M13KE and M13mp18 are significantly different from WT-M13 (P < 0.0001). (B) Phage concentrations of various M13-based clones at 135 minutes of incubation. Each phage clone was amplified separately in an ER2738 culture. At 135 minutes, three aliquots from each flask of growing culture were diluted and plated, and the concentration of phage (pfu/μL) was determined based on plaque counts. The M13KE control was run 21 times for a total of n = 63 platings. WT-M13 was run 6 times (n = 18 platings), M13mp18 and the Ph.D.-7 library were run 4 times each (n = 12 platings), and all other phage clones were run twice each (n = 6 platings). Each bar represents the mean log(pfu/μL) of all platings for a given clone, and the error bars show the 95% confidence interval. Statistical analysis indicated significant differences among the phage concentrations for all the data sets (ANOVA; F_**12,158**_ = 139.1, P < 0.0001). Post-hoc analysis showed that the 135-min concentrations of some clones are significantly different from one another, while others are not (Tukey’s HSD; α = 0.05, see the connecting letters at the bottom of the bars).

A time course for phage amplification was used to compare the rates of propagation among WT-M13, M13mp18, and M13KE (Fig 2A). Each phage clone was used to infect a separate culture of ER2738 cells at the same multiplicity of infection (MOI = 1). Concentrations of the three phage clones, which were monitored at three time points during the 5-hour incubation, were essentially identical at both the beginning (5 × 10^3^ pfu/μL) and end (10^8^–10^9^ pfu/μL), at which point they leveled off at typical concentrations for M13 phage infecting *E*. *coli* cells [43]. A full time course curve would have a sigmoidal shape with the largest difference in concentrations between clones of various propagation rates at 135 minutes, therefore the 135-min time point is sufficient to compare the propagation rates of different clones [40]. At 135 min, both M13mp18 and M13KE had concentrations that were two orders of magnitude lower than that of WT-M13. These results indicate a dramatically lower rate of propagation during the earlier stages of infection for M13 variants containing the lacZα insert.

M13KE-based phage display libraries with variable peptide loops had been constructed at New England Biolabs (K.A. Noren, unpublished). The variable peptide is represented by ACX_*n*_CX_*m*_, where *n* represents the number of randomized residues between two fixed cysteines and *m* represents the number of randomized residues between the second cysteine and a GGGS spacer. A disulfide bond between the two cysteines creates a loop of randomized peptide sequences. Using a mixture of libraries, targetless amplification (2 rounds) and panning against streptavidin (3 rounds) separately yielded several white plaques, suggesting a compromised lacZα gene in these clones. The white plaques were amplified and the phage DNA was purified and sequenced. Two or three clones from each variable loop library were found to have ejected almost the entire lacZα insert, starting twelve nucleotides into the gene. (Note that we use the words “eject” and “ejection” to distinguish this phenomenon from the small or single-nucleotide deletions mentioned elsewhere herein.) The ejection went beyond the lacZα gene, extending 22 nucleotides into Domain B in the origin of replication (ΔlacZα-827 in Fig 1B), ending with position 5890 (position 6705 in M13KE). Thus the phage ejected an 827-nucleotide fragment of the M13KE genome. These clones displayed the peptides ACSYKACWV, ACHYAPCRS, and ACLALACRT from the ACX_4_CX_2_ library, ACRSAGTCP and ACQYAKLCA from the ACX_5_CX_1_ library, and ACNHRLASC and ACSGEERAC from the ACX_6_C library. A slightly larger ejection, extending 33 nucleotides to position 5901 in Domain B (ΔlacZα-838 in Fig 1B), also arose from the ACX_4_CX_2_ library (displayed peptide = ACLMRTCTG).

The propagation rates of the ACX_4_CX_2_ library and its two different ejection clones were evaluated using just the 135-minute time point of the time course experiment described above, providing a snapshot of the middle of the 5 hour incubation (Fig 2B). The ACX_4_CX_2_ library has a phage concentration at 135 minutes that is moderately high compared to that of M13KE, but significantly lower than that of WT-M13. Each of the two ejection clones, ΔlacZα-827 and ΔlacZα-838, has a phage concentration that is not significantly different from WT-M13, suggesting that the ejection of the lacZα gene is sufficient to restore the propagation rate of wild-type phage. Two of the 827-nt ejection clones from the ACX_5_CX_1_ and ACX_6_C libraries (displaying peptides ACRSAGTCP and ACNHRLASC, respectively) also had 135-minute concentrations that were not significantly different from WT-M13 (S1 Fig).

### Fast-propagating M13KE-based clones with single mutations

We returned to an approach we previously reported [41] to identify additional fast-propagating clones from the Ph.D.-7 library. First, three rounds of serial amplification of the phage-displayed peptide library without any panning steps (*i*.*e*., no exposure to a target) allowed fast-propagating clones to be enriched in the amplified pool [40,41]. The next step took advantage of the fact that faster phage have significantly higher concentrations 135 minutes into the infection of *E*. *coli* cells, as illustrated in Fig 2A. The serially amplified library was plated, and randomly selected plaques were used to infect separate 1 mL cultures of *E*. *coli*. At 135 minutes, the cultures were diluted appropriately and plated. The clones with relatively high titers (*e*.*g*., >100 plaques when 10 μL of 10^6^x diluted culture was plated) were identified as Ph-VTAHGGR (G6813A), Ph-SDLVLRP (C6810T), Ph-SRITIDN (G6793T), and Ph-SPTQPKS (G6792C+T5091C), where Ph- represents the phage, the displayed 7-mer peptide follows, and the gene II 5’-UTR mutation is in parentheses. One clone with a relatively high phage concentration, Ph-LMPPPGW, was found to have no mutation in the gene II 5’-UTR. All plaque counts are provided and compared to our previously published data [41] in S2 Table.

The mutations G6813A and C6810T have already been found in clones displaying different peptides, but G6793T and G6792C are mutations that were unknown at the time of our previous publication [41]. We verified that these new mutations confer fast propagation by quantifying phage concentrations 135 minutes into the infection of *E*. *coli*, as described above (Fig 2B). As expected, the concentrations of Ph-SRITIDN (G6793T) and Ph-SPTQPKS (G6792C+T5091C) were significantly higher than those of M13KE and the Ph.D.-7 library, which contains the mixture of all the clones from which Ph-SRITIDN and Ph-SPTQPKS were selected. Neither new mutant clone had a 135-min concentration significantly different from each other or from WT-M13.

Sequencing of the entire genomes of these two new mutant clones revealed no other mutations in Ph-SRITIDN, but the previously observed T5091C mutation in gene IV [41] was found in Ph-SPTQPKS. We isolated the T5091C mutation through a fragment swap between M13KE and Ph-HAFPHLH (G6793Δ + T5091C), a double mutant we discovered previously [41]. The Ph-HAFPHLH fragment contained all of gene IV, including the T5091C mutation, and the HAFPHLH peptide fused to gene III. The M13KE fragment contained the entire 5’-UTR of gene II with no mutation. Thus the recombinant clone Ph-HAFPHLH (T5091C) has just the T5091C mutation along with the displayed peptide HAFPHLH (assumed to be inconsequential). In Fig 2B, the propagation rate of Ph-HAFPHLH (T5091C) is compared to the other fast-propagating clones with 5’-UTR mutations, including Ph-HAFPHLH (G6793Δ+T5091C) and Ph-NoPeptide (G6793Δ). The concentration of T5091C phage at 135 minutes is comparable to both G6793Δ+T5091C and G6793Δ phage, with no statistically significant differences among these three clones. Additionally, Ph-HAFPHLH (T5091C) and Ph-HAFPHLH (G6793Δ+T5091C) propagate just as fast as WT-M13. It is apparent that the T5091C mutation is sufficient to confer fast propagation to M13KE-based phage, but the effects of separate mutations are not additive.

### A designed novel mutation in the gene II 5’-UTR

Point mutations were incorporated into the 5’-UTR of gene II by inserting a 150-bp oligonucleotide between the SwaI and BglII restriction sites in M13KE. The insert was built by polymerase cycling assembly (PCA) [44,45] of six oligonucleotides and was amplified further by PCR. A library of gene II 5’-UTR sequences was incorporated into one of the oligonucleotides by randomizing all 16 positions of the gene II operator sequence 5’-GTTTTTGGGGCTTTTC-3’. The purified insert was ligated into the M13KE cloning vector and the recombinant M13KE/5’-UTR library was used to transfect ER2738 cells. We hypothesized that phage containing advantageous 5’-UTR mutations would not only be viable, but favored in the outgrowth following the transfection of the cells. When plaques from the transfected cell culture were analyzed, most clones were normal M13KE, suggesting that the majority of mutations are deleterious. However, a few clones were found to have 5’-UTR mutations: C6810T, A6809C, T6798Δ, G6793Δ, and G6792T. All of these mutations had already been observed in fast-propagating clones from the Ph.D.-7 and Ph.D.-12 libraries [41]. Sequencing at the peptide library cloning sites within gene III of M13KE confirmed that the newly isolated clones did not contain displayed peptides; therefore, they must have been selected from the 5’-UTR library rather than resulting from a contamination, supporting the effectiveness of a randomized insert to select viable mutants. Still, we have yet to observe any completely novel mutants using this method.

In a modified approach, we created a set of SwaI/BglII inserts that each contained a single 5’-UTR mutation. We designed the mutations G6793C, C6794G, T6795A, T6796G, and T6797C because (i) the same nucleotide position had been found to give rise to other mutations, and/or (ii) the designed mutation would give the 5’-UTR a secondary structure similar to another known mutant, as predicted by the RNA folding function of the mfold web server [46]. Following the transfection of ER2738 with the M13KE/ T6797C ligation, we isolated the new 5’-UTR mutant T6797C that also carried a concomitant deletion, T6789Δ. As demonstrated by Fig 2B, the double mutant T6797C+T6789Δ propagates at a rate significantly faster than M13KE and similar to the other gene II 5’-UTR mutants. To date we have not isolated any novel clones with the remaining four designed mutations.

### Effect of mutations on the propagation of WT-M13

**Fig 3:**
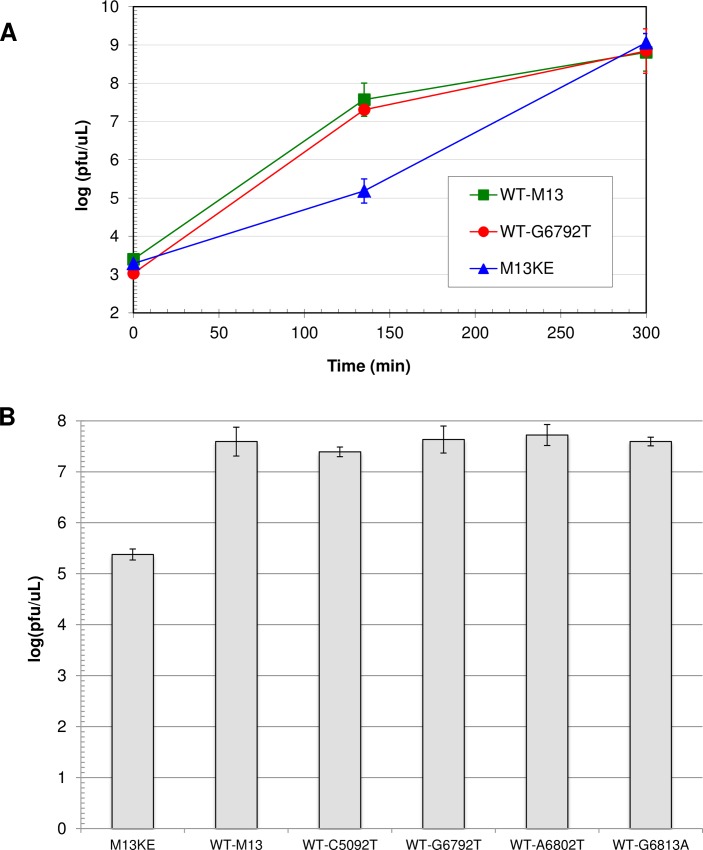
Comparison of propagation rates of wild-type M13 mutants. (A) Time course for amplification of WT-M13, WT-G6792T, and M13KE. Each phage clone was amplified in three separate early log cultures of *E*. *coli* ER2738. From each culture, one aliquot was diluted and plated at the indicated incubation times, and the concentration of phage (pfu/μL) in each growing culture was determined based on plaque counts. Data points represent the mean log(pfu/μL) of the three separate cultures for each type of phage, and the error bars show the 95% confidence interval. Statistical analysis indicated significant differences among the phage concentrations for the three sets of data at 135 minutes (ANOVA; F_**2,8**_ = 318.2, P < 0.0001). Post-hoc analysis showed that WT-M13 and WT-G6792T were not significantly different from each other (Tukey’s HSD; α = 0.05, P = 0.0981). Both WT-M13 and WT-G6792T were significantly different from M13KE (P < 0.0001). (B) Phage concentrations of all WT-M13 mutant clones at 135 minutes of incubation. Each phage clone was amplified separately in an ER2738 culture. At 135 minutes, three aliquots from each flask of growing culture were diluted and plated, and the concentration of phage (pfu/μL) was determined based on plaque counts. The M13KE control was run 12 times for a total of n = 36 platings. WT-M13 was run 6 times (n = 18 platings) and all other phage clones were run twice each (n = 6 platings). Each bar represents the mean log(pfu/μL) of all platings for a given clone, and the error bars show the 95% confidence interval. Statistical analysis indicated significant differences among the phage concentrations for all the data sets (ANOVA; F_**5,77**_ = 239.3, P < 0.0001). Post-hoc analysis showed that M13KE is significantly different from all the other clones (Tukey’s HSD; α = 0.05, P < 0.0001) and that there is no significant difference between any pair among WT-M13 and the WT-mutant clones (range in P = 0.34–1.00).

Single mutations were incorporated into WT-M13 to determine whether they would affect its propagation rate as they do in M13KE-based phage. A standard PCR mutagenesis method was used to amplify the entire genome of WT-M13 with a single mutation incorporated into one of the primers. We chose three gene II 5’-UTR mutations, all of which had arisen spontaneously in the Ph.D. libraries: one in the gene II operator sequence (G6792T), one in the Shine-Dalgarno sequence (G6813A), and one in between these two regions (A6802T). In addition, the mutation C5092T was constructed because it is the opposite of the T5091C mutation in M13KE. None of the mutations has an effect on the propagation rate of WT-M13; each mutant clone has essentially the same 135-min phage concentration as the wild-type phage (Fig 3).

## Discussion

### An insert in the M13 replication enhancer decreases the propagation rate of the phage

The M13 (+) strand origin is comprised of two key domains (Fig 1A). Domain A (or the “core origin,” positions 5769–5819) is required for both (+) strand initiation and termination, and Domain B (position 5820 to ~5910) is only required for initiation [20]. According to our data, M13mp18 and M13KE propagate at considerably slower rates than WT-M13 (Fig 2A), suggesting that the interruption of Domain B between positions 5868 and 5869 by the lacZα gene hinders the replication of viral DNA during the life cycle of the phage. Domain B is the replication enhancer, which houses the binding site for *E*. *coli* integration host factor (IHF) [47]. IHF is known to enhance DNA replication in numerous contexts [48–50]. In Ff phage, IHF is an activator of, but not a requirement for, viral DNA replication [47]. The heterodimer binds in Domain B primarily at a site spanning positions 5825–5857 and, independently and less strongly, at 5886–5942. An infected *E*. *coli* strain lacking IHF still produces f1 phage, but at a rate only 3% of that observed in a normal strain containing IHF. Rather, when IHF is expressed, but deletions are made in Domain B at positions 5820–5836 and 5821–5850, which include a large part of the primary IHF binding site, biological activity is reduced to only 1% (calculated from the transduction of antibiotic resistance by packaged single-stranded pBR322 harboring the M13 functional origin compared to a control plasmid) [20]. In addition, the insertion of 8 and 16 nucleotides between positions 5829 and 5830 reduces biological activity to 30% and 1%, respectively. Johnston and Ray similarly constructed a series of M13 deletion mutants lacking sections of Domain B in the range 5813–5850 [31]. While all deletion mutant phages were still produced, both the rate of phage production and single-stranded viral DNA synthesis were reduced with increased length of deletion. All these findings suggest that optimal binding of Domain B by IHF is required for the most effective employment of the *ori* in phage replication. However, with either a compromised binding site or the absence of IHF itself, replication still occurs, albeit at a reduced efficiency (see more discussion of the role of IHF below). In M13mp-based phage, the lacZα gene is inserted between the two binding regions of IHF–eleven nucleotides downstream of the stronger binding site and seventeen nucleotides upstream of the weaker one. Although IHF has been found to bind its two sites independently [47], it may be that IHF binding is compromised when the two sites are vastly separated, which would explain the reduced propagation rates we observe for M13mp18 and M13KE.

There are several other differences between M13mp18/M13KE and WT-M13 that are outside of the lacZα gene (S1 Table), but it is probable that the large insert is the primary cause of the reduced propagation. Other genetic differences may either magnify or compensate for the replication defect. For example, Zinder and coworkers discovered the G6125T mutation in gene II of Messing’s M13mp1 phage, which as helper phage was able to rescue chimeric plasmids lacking Domain B in the f1 functional origin [51]. This Met40Ile mutation in gIIp has been passed down to M13mp18 and M13KE, but does not appear to fully compensate for the lacZα insert in our propagation assay. It is not clear whether the discrepancy lies in the different assay used or in the particular M13mp phage characterized, but our time-based phage titer clearly demonstrates that the later generations of M13mp-based phage still have a propagation disadvantage compared to WT-M13, despite G6125T and other mutations in the genome.

### Wild-type M13 propagation is recovered through the ejection of the lacZα gene

A rather plausible mechanism by which M13KE might recover fast propagation would be the simple ejection of the lacZα insert that interrupts Domain B of the *ori*. Remarkably, the vast majority of M13KE-based phage retain the lacZα gene, as evidenced in the extensive use of the Ph.D. libraries without significant occurrence of white plaques caused by phage lacking the insert. During the construction of a series of constrained-loop variable peptide libraries, several clones were found to have ejected almost the entire lacZα insert, but the ejection was not entirely clean (Fig 1B). The first eleven nucleotides of the lacZα gene remained as a scar. In addition, either 22 or 33 nucleotides of Domain B were deleted along with most of the gene. The smaller ejection, ΔlacZα-827, arose from libraries of all three different loop sizes, ACX_4_CX_2_, ACX_5_CX_1_, and ACX_6_C, suggesting a possible mutational hotspot. However, only one library, ACX_4_CX_2_, produced a clone with a larger ejection, ΔlacZα-838. According to our propagation assay, both ejections effectively restore the propagation rate of WT-M13, and the two deletions cannot be distinguished from each other in this respect (Fig 2B). Neither ejection includes any part of the stronger IHF binding site. However, the smaller (5869–5890) and larger (5869–5901) ejections remove five and sixteen nucleotides, respectively, of the 57-nt weaker IHF binding site. These findings suggest that the entirety of the secondary binding site is less important than the proximity of the two sites, which is restored upon the ejection of the lacZα insert. This compensatory mechanism is reminiscent of the findings of Smith and coworkers, in which an fd minus stand origin interrupted by the tetracycline resistance gene was spontaneously restored by a rearrangement that moved the Tet gene to a site outside the minus stand origin [52].

### Wild-type M13 propagation is recovered through gene II 5’-UTR mutations

**Fig 4:**
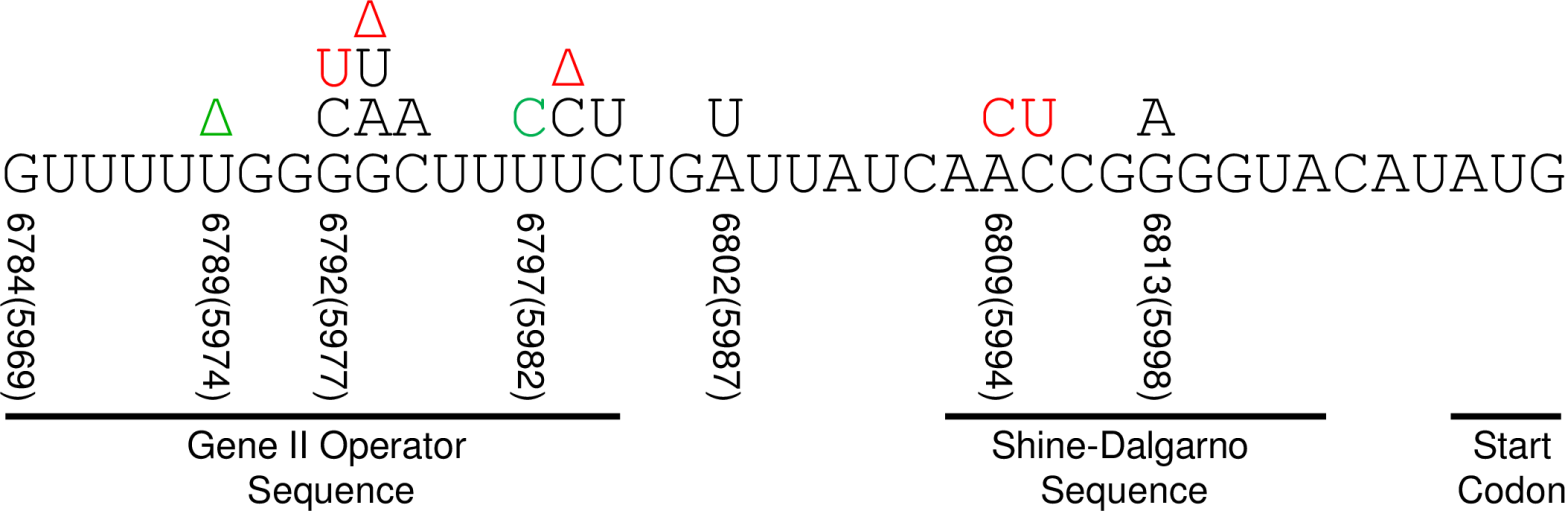
Mutations in the 5’-UTR of gene II mRNA in M13KE. The normal 5’-UTR region of the mRNA is shown, containing the gene II operator sequence, the Shine-Dalgarno sequence, and the start codon. The nucleotide numbering corresponds to M13KE. Thirteen different spontaneous 5’-UTR mutations are indicated above their respective nucleotides within the 5’-UTR (black and red). The majority of these mutations were previously reported [41], and were discovered in one of five ways: (i) in Ph.D.-7 or Ph.D.-12 phage display experiments that used Zn^2+^ as a target, (ii) during serial amplification of the Ph.D.-7 library, (iii) in a 135 minute screen of amplified Ph.D.-7 or Ph.D.-12 libraries, (iv) during amplification of M13KE, or (v) as a clone that contaminated a concurrent experiment in our lab. The mutations G6793T and G6792C are new to this publication, and were discovered via method (iii). The T6797C mutation was synthetically introduced using an insert containing the mutation, and was accompanied by a concomitant deletion, T6789Δ (both green). The mutations shown in red were made using a randomized synthetic insert, but had already arisen spontaneously in our experiments. Several mutations have been discovered repeatedly with different displayed peptides (or no peptide), including the two clones reported herein, Ph-VTAHGGR and Ph-SDLVLRP, which have new peptides but previously found mutations. These repeated mutations are listed here as: Mutation (Number of times observed): G6813A (4), C6810T (2), A6809C (3), T6798C (4), T6798Δ (3), G6793A (3), G6793Δ (2), G6792T (4). Two additional mutations arose through method (i) and are not shown here because they are upstream of the mRNA sequence: G6748Δ (in the gene II promoter) and C6589T (in the lacZα insert) [41].

In 2014, we reported a repertoire of single mutations and deletions in the region upstream of gene II, the vast majority of which are in the untranslated region between the 5’-end of the mRNA and the start codon for gene II [41]. Each mutation or deletion conferred fast propagation to the M13KE-based phage clone in which it was found. We first identified Ph-HAIYPRH (G6813A) during phage display experiments using the Ph.D.-7 library [40]. We suspected that HAIYPRH was a target-unrelated peptide [53] that appeared repeatedly in our results due to a propagation-related [52] advantage possessed by the phage clone on which it was displayed. Subsequently, we were able to use the fast-propagating phenotype to find additional mutant phage in serially-amplified pools of the Ph.D.-7 library, the Ph.D.-12 library, and even M13KE (no displayed peptide) [41]. One screening method targeted phage clones that had the highest phage concentrations after a 135-minute infection of *E*. *coli*. Here, we further mined the same batch of amplified Ph.D.-7 library and discovered two clones carrying completely new 5’-UTR mutations, Ph-SPTQPKS (G6792C+T5091C) and Ph-SRITIDN (G6793T). In our standard phage propagation assay, each of these clones has a propagation rate that is similar to WT-M13 (Fig 2B). We also identified two new clones carrying mutations we had already discovered: Ph-VTAHGGR (G6813A) and Ph-SDLVLRP (C6810T). In addition to being the first reported 5’-UTR mutation, G6813A is one of the most recurrent mutations (*i*.*e*., it has been found in four different clones from both the Ph.D.-7 and Ph.D-12 libraries) and confers to the phage among the fastest propagation rates [41]. In contrast, the C6810T mutation is less common; it has been observed in just one other clone, Ph-HQLHHHL. In total, we have discovered thirteen unique spontaneous mutations or deletions in the gene II 5’-UTR (Fig 4), in addition to one mutation and one deletion that are upstream of this region and therefore not part of the mRNA sequence (see full list in S3 Table). All fifteen mutations/deletions confer a phage propagation rate that is significantly higher than that of M13KE, and which approaches or equals that of WT-M13 [41].

A balance among the filamentous phage proteins is critical in maintaining a persistent infection that does not significantly disturb the metabolism of the infected cells; in particular, the regulation of gIIp activity is important in controlling replication [29,30,54–57]. The 5’-UTR of gene II (Fig 4) is the regulatory region for the translation of the gene into the initiator protein. It includes the Shine-Dalgarno (SD) sequence, which is the primary binding feature recognized by the ribosome [58,59]. Translation initiation relies on the recruitment of the ribosome to bind at and around the SD sequence on the mRNA. Additionally, the expression of gene II is regulated by the repressor protein gVp [29,30], which binds the 5’-UTR at the operator sequence. Thus, the degree to which gIIp is produced depends on the binding of both the ribosome to the SD sequence and gVp to the operator sequence. While the 5’-UTR nucleotide sequence certainly influences these two binding events, it is likely that the secondary structure of the mRNA also has a significant effect [60–65]. Despite some controversy, there is compelling evidence to suggest that the mRNA forms a stable structure [56,66–68]. A mutation or deletion anywhere in the 5’-UTR has the potential to alter the secondary structure of the mRNA in such a way as to enhance ribosome binding and/or inhibit gVp binding, both of which result in increased gIIp synthesis. Zinder and coworkers indeed demonstrated that mutations in the gene II operator sequence lowered the binding affinity of gVp for its target site [69], effectively increasing the amount of gIIp produced [51,66]. Five of our M13KE mutations, including the recently discovered G6793T in Ph-SRITIDN, correspond to Zinder’s mutations in the gene II operator sequence of f1 phage [66]. However, we have also observed several mutations that have not been found in f1, including the new mutation G6792C in Ph-SPTQPKS and both the mutation and deletion in T6797C+T6789Δ. Additionally, we have previously found mutations downstream of the gene II operator, before and within the SD sequence [41]. The prevalence of advantageous mutations throughout the 5’-UTR, from the gene II operator through the SD sequence, suggests that changes to the primary and/or secondary structure of this regulatory region result in enhanced gene II expression, which in turn compensates for the lacZα insert in the replication enhancer of the M13mp-derived vectors.

Further evidence indicates that a defective (+) stand origin can be overcome by other changes to gIIp that are either quantitative, as described above, or qualitative. In the former case, an alternative mechanism by which gIIp is overproduced is through functional changes to gVp that reduce its activity as a repressor. Various Arg→Cys mutations in gVp have been shown to decrease its binding to the mRNA, increase the production of gIIp, and make Domain B completely dispensable [51,66,70]. Rather, in a qualitative manner, mutations in the N-terminal portion of gIIp itself (including the Met40Ile mutation in M13mp1) do not affect its intracellular concentration, but nevertheless restore replication initiation in the absence of either IHF or Domain B through a presumed change in activity [47,51,70,71]. Some gIIp mutants were characterized by an enhancement in the cooperative binding of the RF I recognition sequence [72] or a lessened requirement for supercoiling in order to nick the genome [73]. Still, it remains unclear how increased production of gIIp or mutations within it compensate for an impaired replication enhancer. For instance, gIIp and IHF bind to RF I independently in Domains A and B, respectively [47]. Moreover, *in vitro* nicking of the double-stranded genome by wild-type gIIp is not compromised by the absence of either Domain B [20] or IHF [73]. It has been speculated that subsequent to nicking, a key interaction takes place in Domain B for which either more concentrated or mutant gIIp loosens the specificity [73,74]. Both IHF and gIIp have been found to bend the replication origin as part of their respective binding events, suggesting that a bent or unwound DNA complex including both proteins is important in replication initiation [47]. IHF may enhance the activity of gIIp in unwinding the RF I DNA and forming the replication fork [47,70,73]. It is also possible that phenomena occurring *in vivo* have not been simulated in some of the *in vitro* studies described [73].

Gene II 5’-UTR mutations are selected to compensate for the replication defect, as evidenced by the fact that G6792T, A6802T, G6813A do not enhance phage propagation in WT-M13 (Fig 3). When replication is already optimized, as in wild-type phage, an increase in the intracellular concentration of gIIp may not alter the rate of phage propagation. Indeed, Zaman *et al* demonstrated that the deletion of almost the entire gene II operator sequence had no effect on the viability of M13 phage, even when the host cells were grown on minimal medium [57]. In a systematic study of single-base mutations scattered throughout the f1 genome, Peris *et al* found only 2/100 mutations (C3748A = Glu185Lys in gIp and T4541A = Ser108Thr in gIVp) to be statistically beneficial to the phage [75]. The 7% enhancement in the rate of phage amplification (calculated from the change in the log phage titer over time) would correspond to only a 0.2 increase in the log(pfu/μL) at 135 minutes in our propagation assay, which would not constitute a significant difference. It is possible that with a more sensitive assay, we might ascertain slightly higher propagation rates for the WT-M13 mutants, but the rate enhancement would be negligible compared to the ~230% increase that results from mutations in M13KE. It is apparently the replication defect caused by the lacZα insert that provides the opportunity for increased gIIp concentration to have a significant impact on the rate of replication.

### Occurrences and properties of point mutations and deletions

The fifteen compensatory mutations in M13KE-based phage are represented across 32 different clones, which have various peptides displayed on gIIIp or, in a few cases, no displayed peptide at all (S3 Table). Seven mutations have occurred only once, but eight mutations have arisen in two or more unique clones (see Fig 4 caption). We have discussed previously how phage clones possessing the most recurrent mutations generally (i) have the fastest propagation rates, (ii) have relatively high abundances in the naïve Ph.D. libraries from which they come, and (iii) tend to be reported in the literature and appear in databases for their appearances in phage display experiments [41]. Through these three lenses, we analyzed the two new clones with unprecedented mutations, Ph-SPTQPKS (G6792C+T5091C) and Ph-SRITIDN (G6793T). With an average 135-min phage concentration of log(pfu/μL) = 7.19, Ph-SPTQPKS falls in the middle of the range of concentrations observed for all the mutant phage clones (S3 Table and [41]). In contrast, Ph-SRITIDN (log(pfu/μL) = 7.39) approaches the high end of the range that includes Ph-HAIYPRH (G6813A, log(pfu/μL) = 7.45) and Ph-GKPMPPM (G6792T, log(pfu/μL) = 7.69). In fact, all of these clones propagate at rates that are statistically equivalent to that of WT-M13 (log(pfu/μL) = 7.59), and are distinct from the roughly one fourth of all the 5’-UTR mutants that are significantly faster than M13KE, but not quite as fast as WT-M13 [41].

In the construction of the Ph.D. libraries, the ligation of the randomized peptide-encoding oligonucleotide between the KpnI and EagI sites in M13KE was followed by an amplification step. The propagation advantage of clones carrying certain mutations/deletions presumably allowed these phage to become enriched in the library during the first amplification step. The existence of Ph.D. library mutants that both have and lack displayed peptides suggests that spontaneous changes arose during library construction, but also existed in a small fraction of the M13KE library vector [41]. Deep sequencing of 4 × 10^6^ clones from the naïve Ph.D.-7 library by Derda and coworkers showed that, while >99% of the population was expected to have a single copy number (abundance = 2.5 × 10^−7^), about 28% of the peptide sequences had higher than theoretical abundances [76]. Based on this analysis, we reported that approximately 1/3 of our fast-propagating phage clones had relatively high abundances in the naïve Ph.D.-7 or Ph.D-12 libraries, ranging from 8 × 10^−7^ to 0.0014 (abundance = number of occurrences divided by 4 × 10^6^ clones) [41]. The new clone Ph-SPTQPKS has a very high abundance of 0.00045, ranking it third after our two most abundant mutant clones, Ph-GKPMPPM (abundance = 0.0014) and Ph-HAIYPRH (abundance = 0.00053). Ph-SRITIDN was not found to have enriched abundance in the naïve library, akin to an approximate 2/3 majority of all the mutant clones we have analyzed. It is not clear why some of the fast-propagating phage clones were enriched upon library construction, while others were not, despite similar behavior in our propagation assay.

During a typical phage display experiment, three to four rounds of panning each involves exposing the phage-displayed peptide library to the target of interest. Following all but the last round of panning, the phage pool is amplified in *E*. *coli* culture. Each amplification provides an opportunity for fast-propagating phage to become enriched in the pool. In our first report of Ph-HAIYPRH (G6813A), we showed a very high abundance of >0.1 among the plaques analyzed after three rounds of targetless serial amplification of the Ph.D.-7 library (14 out of 134 plaques were Ph-HAIYPRH) [40]. Additionally, when equivalent MOI of Ph-HAIYPRH and the Ph.D.-7 library were combined in the same *E*. *coli* culture, Ph-HAIYPRH completely took over the culture within 90 minutes. Derda’s library sequencing method described above demonstrated that after only one round of amplification, abundances increased significantly for Ph-GKPMPPM (0.0014 → 0.012), Ph-HAIYPRH (0.00053 → 0.013), and Ph-SPTQPKS (0.00045→0.0069) [76]. The propensity for certain clones to become enriched during amplification explains why the “target-unrelated peptides” displayed on such phage may abound in the results of panning experiments, even when the peptides have a low affinity for the target. However, unlike Ph-HAIYPRH, Ph-GKPMPPM, and several other 5’-UTR mutant clones [41], neither Ph-SRITIDN nor Ph-SPTQPKS has been reported in the results of phage display experiments employing the same lot of the Ph.D.-7 library. This fact is much less surprising for the former than the latter, which has a particularly high abundance in the naïve Ph.D.-7 library.

WT-M13 and all the M13mp vectors including M13mp18 and M13mp19 contain a C at position 5091. M13KE alone has a T in this position, leading to a silent mutation in gene IV (GTC→GTT, Val 291). The mutation most likely occurred during the construction of M13KE, when Kunkel mutagenesis was used to incorporate the KpnI and EagI restriction sites into M13mp19. In the first step, the template for mutagenesis was passed through the *dut*, *ung* strain CJ236, and we suspect that an unrepaired cytosine deamination lesion led to a C→T mutation at position 5091 (due to the lack of uracil-DNA glycosylase activity in the *ung* strain). Approximately one third of all the 5’-UTR mutations and deletions in M13KE occur concomitantly with a T5091C mutation, which is a reversion back to the original wild-type base (5092C in WT-M13). In double-mutant clones, the T5091C mutation does not appear to further enhance propagation beyond the effect of the 5’-UTR mutation; a clone containing the same 5’-UTR mutation, but lacking the T5091C mutation, propagates equally fast [41]. We sought to determine whether an isolated T5091C mutation would have any effect on phage propagation. When we removed the G6793Δ deletion from the double mutant Ph-HAFPHLH (G6793Δ+ T5091C), the single mutant Ph-HAFPHLH (T5091C) was found to propagate at a similar rate to both the double mutant and a clone containing just the G6793Δ deletion (Fig 2B). Thus the T5091C mutation appears to be sufficient to confer fast propagation to M13KE phage. It is curious that M13mp18, which has the wild-type C at position 5091, does not propagate at the faster rate. It also remains elusive how a silent mutation in gene IV–indeed, a reversion to the wild-type nucleotide–can affect phage propagation. In fact, WT-M13 does not require a C at this position; when it was replaced by T in WT-C5092T, the propagation rate was not affected (Fig 3B). The 5091 mutation is seemingly unrelated to the defect posed by the lacZα insert, and it likely has a different influence on phage propagation that may be related to the role of gIVp in phage assembly and export [77]. Although the mutation is silent (the wobble position of a pair of common valine codons), it may affect the level of gIVp expression and folding in a subtle way that invites future study.

Prior to this manuscript, we suspected we had already found most, if not all, of the spontaneous gene II 5’-UTR mutations capable of increasing the propagation rate of M13KE. The 28 different clones we had discovered carried only 13 unique mutations, and we were indeed rather surprised to find two unique mutations among the four new clones described above. We then sought to create novel mutations synthetically by selecting viable phage from a library of clones randomized throughout the 16-nt operator sequence [66,69]. The only mutations/deletions that were derived from this 5’-UTR library overlapped with spontaneous ones from the Ph.D. libraries: C6810T, A6809C, T6798Δ, G6793Δ, and G6792T. All five mutations/deletions had been found in at least two Ph.D. phage clones, suggesting a proclivity of M13KE to incorporate these particular genetic changes. To date, two of the most recurrent spontaneous mutations, G6813A and T6798C, have not been obtained from the 5’-UTR library. We suspect that with more extensive probing of the randomized 5’-UTR, we might encounter these and other familiar mutations. Based on our findings, we are pessimistic about the effectiveness of this method in selecting novel mutant clones, which would have to compete with more robust mutants such as G6792T following the transfection of cells with the recombinant M13KE vector.

We consequently aimed to engineer individual mutations into M13KE by replacing the 5’UTR with an insert modified at only one position at a time. Although the exact nature of gene II mRNA folding is unknown [58,68–70], we used predicted secondary structures to roughly guide the design of novel mutations. The RNA folding function of the mfold web server [46] was used to predict the lowest energy mRNA secondary structure for each of the known 5’-UTR mutations/deletions (see S2 Fig for structures and ΔG_fold_ values). Eight mutations/deletions gave essentially the same stem-loop structure as the normal 5’-UTR sequence in M13KE, while five had various other structures. Interestingly, all but one were predicted to have the same or greater stability than the normal sequence based on the calculated free energy of folding (ΔG_fold_). Assuming that secondary structure influences the expression of gene II, as described above, we chose to incorporate a T6797C mutation because its mRNA was predicted to have a stem-loop structure somewhat similar to the highly recurrent spontaneous mutation G6792T [41]. We successfully obtained the mutant T6797C, but it also contained T6789Δ, a deletion of one of the Ts in the five-T stretch near the 5’ extreme of the UTR (Fig 4). The deletion likely arose spontaneously during either PCA of the insert or its subsequent amplification by PCR. No mutations or deletions in this stretch of the 5’-UTR have arisen spontaneously from replication-defective phage, but T→A and T→C mutations selected from a randomized 5’-UTR library were found to decrease repression of gene II by 50% [66]. Interestingly, the T6789Δ deletion does not alter the predicted secondary structure of the mRNA beyond the effect of the T6797C mutation (S2 Fig). More importantly, T6797C+ T6789Δ phage has a propagation rate similar to the other 5’-UTR mutants (Fig 2B). Although we have not yet synthesized any other designed mutations, this example demonstrates that it is possible to predict and engineer compensatory 5’-UTR mutations that have not arisen spontaneously from the phage display libraries.

M13KE phage has the capacity to eject the lacZα insert or pick up point mutations and deletions in order to recover the propagation rate of wild-type phage. However, the M13KE-based phage in the Ph.D. libraries is generally very stable, as evidenced by the vast majority of clones that are neither overrepresented in the library nor characterized by mutations. In phage display experiments, careful selection of blue plaques is critical, as white plaques send a clear signal that the lacZα gene has been compromised. It is always possible that blue plaques contain phage mutants that have been enriched during panning due to a propagation advantage. This phenomenon is thought to be particularly likely when the target has a weak affinity for the peptides in the library [41]. However, the existence of propagation-enhanced clones does not exclude the selection of authentic target-binders. Thorough verification assays for target-binding and careful sequencing, particularly in mutational hot spots, allow the effective identification of useful ligands by phage display even in the presence of clones harboring advantageous mutations.

## Supporting information

S1 Table Outline of genetic differences among WT-M13, M13mp18, and M13KE. Genomic sequences were obtained from our own sequencing (WT-M13) or that previously performed at New England Biolabs (M13mp18 and M13KE). Nucleotide numbers in the leftmost column are for WT-M13. A nucleotide is underlined when it differs from the genome to its left in the table. Differences between M13mp18 and M13KE in the polylinker, which is reversed in M13KE and missing the KpnI site, are not indicated. Codon assignments are based on van Wezenbeek *et al* [17].
^a^ In M13mp18 and M13KE, the nucleotide number would be lower by 1 nt compared to WT-M13 due to the deletion of 1565T.
^b^ In M13mp18, the nucleotide number would be higher by 842 nt compared to WT-M13 due to the lacZα insert.
^c^ In M13KE, the nucleotide number would be higher by 815 nt compared to WT-M13 due to the lacZα insert, which contains a shorter polylinker than in M13mp18.
^d^ Mutations made to M13mp19 to incorporate the KpnI and EagI cloning sites into M13KE.
(DOCX)

S2 Table 135-minute screens of the serially amplified (round 3) Ph.D.-7 and Ph.D.-12 libraries. Fast-propagating clones were identified from the amplified libraries as previously described [41]. Briefly, twelve or more plaques from the third round of serial amplification were each used to infect 1 mL of early log ER2738 culture. Each value given in the table is the number of plaques obtained when 10 μL of diluted phage-infected *E*. *coli* culture were plated at 135 minutes of incubation. The plaques from clones with particularly high numbers were amplified to purify the viral DNA, and the clones were identified by sequencing both the gene II 5’-UTR and the displayed peptide fused to gene III. The clones in the rows shaded in gray are from the current publication. All other clones were reported by Nguyen *et al* [41]. These data, which derive from the infection of relatively small cell cultures by inexact numbers of virions from a plaque, serve as an approximate measure of phage propagation; they should not be confused with the more precise phage propagation assays represented in Figs 2 and 3 and S3 Table. One column indicates clones that were also sequenced at position 5091: Y means that the T5091C mutation is present, N means that there is no T5091C mutation, and ND means that the clone was not sequenced at position 5091.
(DOCX)

S3 Table Summary of spontaneous mutations in the gene II 5’-UTR of M13KE. In the normal M13KE sequence, the Shine-Dalgarno (SD) region is in bold, the gene II RNA operator sequence is italicized+bold, and the start codon is underlined. Phage clones are named according to the displayed peptide; some phage clones in the table have no peptide insert as indicated. The summary includes clones reported in Nguyen *et al* [41] as well as in this publication. Mutant clones were discovered as follows: ^a^ in Ph.D.-7 or Ph.D.-12 phage display experiments using Zn^2+^ as the target, ^b^ in serial amplification of the Ph.D.-7 library, ^c^ in a 135-minute screen of the serially amplified Ph.D.-7 or Ph.D.-12 library, ^d^ in the amplification of M13KE, ^e^ as a contaminating clone in a concurrent experiment in our lab. One column indicates clones that were also sequenced at position 5091: Y means that the T5091C mutation is present (also indicated by an asterisk * next to the name of the clone), N means that there is no T5091C mutation, and ND means that the clone was not sequenced at position 5091. The log(pfu/μL) at 135 minutes of incubation with *E*. *coli* cells are the same values plotted in the graphs of the respective publications (results corresponding to Fig 2B of the current work are underlined; results from Nguyen *et al* are not underlined; ND = not determined). Differences between old and new data for the same clone are due to small variations in conditions when experiments are performed on separate occasions, and they are generally not statistically significant. Each “x” indicates that the peptide has been identified in panning experiments reported in the literature and/or is present in the databases MimoDB, SAROTUP, and/or PhD7Faster, which is part of the SAROTUP suite (see details in the Discussion of Nguyen *et al* [41]). The last column contains the abundances of certain clones in the naïve library determined through deep sequencing (number of occurrences divided by 4 x 10^6^ clones analyzed) as reported by Derda and coworkers [76]. Clones without abundances were not detected in Derda’s study.
(DOCX)

S1 Fig Concentrations at 135 minutes of amplification of phage from constrained loop libraries. One ΔlacZα-827 clone arose spontaneously from the ACX_5_X_1_ library and the other arose spontaneously from the ACX_6_C library, and are indicated as such. Each phage clone was amplified separately in an ER2738 culture. At 135 minutes, three aliquots from each flask of growing culture were diluted and plated, and the concentration of phage (pfu/μL) was determined based on plaque counts. The M13KE control was run 16 times for a total of n = 48 platings. WT-M13 was run 6 times (n = 18 platings), and all other phage clones were run twice each (n = 6 platings). Each bar represents the mean log(pfu/μL) of all platings for a given clone, and the error bars show the 95% confidence interval. Statistical analysis indicated significant differences among the phage concentrations for all the data sets (ANOVA; F_5,89_ = 212.3, P < 0.0001). Post-hoc analysis showed that the 135-min concentration for the ΔlacZα-827 ejection from the ACX_5_CX_1_ library is significantly higher than that of the library from which it arose (Tukey’s HSD; α = 0.05, P < 0.0001). Similarly, the ΔlacZα-827 ejection from the ACX_6_C library is significantly higher than its library (P < 0.0001). Both ejections are not statistically different from WT-M13 (P > 0.85).
(PPTX)

S2 Fig Secondary structures of the gene II 5’-UTR in the presence and absence of mutations as predicted by mfold. Sequences of the gene II 5’-UTR through the start codon were entered in the RNA folding form on the mfold web server [46]. All default parameters were used. In each case, the Structure 1 pdf was opened, and the image was copied into this document. Mutations and deletions are indicated by yellow circles and stars, respectively. The mutations shown have arisen spontaneously from the Ph.D.-7 and Ph.D.-12 libraries or from M13KE, with the exceptions of U6797C and U6797C+U6789Δ. We attempted to synthetically incorporate U6797C into M13KE based on its similar secondary structure to G6792U. We instead obtained U6797C+U6789Δ, which has a predicted secondary structure that is essentially the same as that predicted for U6797C. ΔG_fold_ is the free energy change for folding.
(PPTX)
