# Supplementary material for: Various mutations compensate for a deleterious lacZα insert in the replication enhancer of M13 bacteriophage
Source: PLoS One. 2017 Apr 26;12(4):e0176421. doi: 10.1371/journal.pone.0176421 (PMC5405960; doi:10.1371/journal.pone.0176421)
Supplement: S1 Table — Genomic sequences were obtained from our own sequencing (WT-M13) or that previously performed at New England Biolabs (M13mp18 and M13KE). Nucleotide numbers in the leftmost column are for WT-M13. A nucleotide is underlined when it differs from the genome to its left in the table. Differences between M13mp18 and M13KE in the polylinker, which is reversed in M13KE and missing the KpnI site, are not indicated. Codon assignments are based on van Wezenbeek et al [17]. a In M13mp18 and M13KE, the nucleotide number would be lower by 1 nt compared to WT-M13 due to the deletion of 1565T. b In M13mp18, the nucleotide number would be higher by 842 nt compared to WT-M13 due to the lacZα insert. c In M13KE, the nucleotide number would be higher by 815 nt compared to WT-M13 due to the lacZα insert, which contains a shorter polylinker than in M13mp18. d Mutations made to M13mp19 to incorporate the KpnI and EagI cloning sites into M13KE. (DOCX) [file pone.0176421.s001.docx]

| **WT-M13 numbering** | **WT-M13** | **M13mp18** | **M13KE** | **Description of Mutation** |
| --- | --- | --- | --- | --- |
| 3  149  213  214,216  1229  1565  1611  1614  1632  1635  1663  2224,2225  2229  5092  5868*--lacZα--*5869  5909  6095  6125 | C  C  C  C,G  C  T  T  T  C  T  C  C,C  C  C  C  C  G | T  T  T  T,T  T  Δ  T^a^  T^a^  C^a^  T^a^  T^a^  T^a^,T^a^  T^a^  C^a^  T^b^  T^b^  T^b^ | T  T  T  T,T  T  Δ  G^a,d^  A^a,d^  G^a,d^  C^a,d^  T^a^  T^a^,T^a^  T^a^  T^a^  T^c^  T^c^  T^c^ | Gene II: AAC🡪AAT (Asn135,silent)  Gene II: ACA🡪ATA (Thr184Ile)  Gene II: CAC🡪CAT (His205,silent)  Gene II: CAG🡪TAT (Gln206Tyr)  Gene IX: TTC🡪TTT (Phe8,silent)  Gene VIII/Gene II IG region  Gene III: GTT🡪GTG (Val11,silent)  Gene III: GTT🡪GTA (Val12,silent)  Gene III: TCC🡪TCG (Ser18,silent)  Gene III: GCT🡪GCC (Ala19,silent)  Gene III: CCC🡪TCC (Pro29Ser)  Gene III: CCA🡪TTA (Pro216Leu)  Gene III: TTC🡪TTT (Phe217,silent)  Gene IV: GTC🡪GTT (Val291,silent)  Gene IV/Gene II IG region (ori)  Gene II: GAC🡪GAT (Asp30,silent)  Gene II: ATG🡪ATT (Met40Ile) |
