# Supplementary material for: Various mutations compensate for a deleterious lacZα insert in the replication enhancer of M13 bacteriophage
Source: PLoS One. 2017 Apr 26;12(4):e0176421. doi: 10.1371/journal.pone.0176421 (PMC5405960; doi:10.1371/journal.pone.0176421)
Supplement: S2 Table — Fast-propagating clones were identified from the amplified libraries as previously described [41]. Briefly, twelve or more plaques from the third round of serial amplification were each used to infect 1 mL of early log ER2738 culture. Each value given in the table is the number of plaques obtained when 10 μL of diluted phage-infected E. coli culture were plated at 135 minutes of incubation. The plaques from clones with particularly high numbers were amplified to purify the viral DNA, and the clones were identified by sequencing both the gene II 5’-UTR and the displayed peptide fused to gene III. The clones in the rows shaded in gray are from the current publication. All other clones were reported by Nguyen et al [41]. These data, which derive from the infection of relatively small cell cultures by inexact numbers of virions from a plaque, serve as an approximate measure of phage propagation; they should not be confused with the more precise phage propagation assays represented in Figs 2 and 3 and S3 Table. One column indicates clones that were also sequenced at position 5091: Y means that the T5091C mutation is present, N means that there is no T5091C mutation, and ND means that the clone was not sequenced at position 5091. (DOCX) [file pone.0176421.s002.docx]

| **Amplified Library** | **# Plaques**  **(10^5^ dilution)** | **# Plaques**  **(10^6^ dilution)** | **5’-UTR Mutation** | **T5091C**  **?** | **Peptide** |
| --- | --- | --- | --- | --- | --- |
| Ph.D.-7 | 9 | 1 | Normal (negative control) | ND | LRTDPIF |
| Ph.D.-7 | 29 | 2 | Normal (negative control) | ND | No Peptide |
| Ph.D.-7 | 641 | 85 | A6802T | Y | SNHAPRH |
| **Ph.D.-7** | **1012** | **130** | **Normal** | ND | **LMPPPGW** |
| **Ph.D.-7** | **1044** | **135** | **C6810T** | ND | **SDLVLRP** |
| **Ph.D.-7** | **1188** | **130** | **G6793T** | N | **SRITIDN** |
| **Ph.D.-7** | **1404** | **237** | **G6813A** | ND | **VTAHGGR** |
| **Ph.D.-7** | **1580** | **296** | **G6792C** | Y | **SPTQPKS** |
| Ph.D.-7 | 2008 | 174 | G6792T | N | GKPMPPM |
| Ph.D.-7 | 2208 | 253 | T6798C | ND | ARPPASP |
| Ph.D.-12 | 24 | 3 | Normal (negative control) | ND | VFCTTPQRPITS |
| Ph.D.-12 | 1536 | 103 | A6809C | ND | No Peptide |
| Ph.D.-12 | 1616 | 146 | C6799T | ND | HEASQHAFSARL |
| Ph.D.-12 | 2336 | 220 | G6792T | ND | KDTNIYDQRYSR |
| Ph.D.-12 | 3216 | 250 | T6795Δ | N | AMSPRMDGKVFA |
