# Supplementary material for: Various mutations compensate for a deleterious lacZα insert in the replication enhancer of M13 bacteriophage
Source: PLoS One. 2017 Apr 26;12(4):e0176421. doi: 10.1371/journal.pone.0176421 (PMC5405960; doi:10.1371/journal.pone.0176421)
Supplement: S3 Table — In the normal M13KE sequence, the Shine-Dalgarno (SD) region is in bold, the gene II RNA operator sequence is italicized+bold, and the start codon is underlined. Phage clones are named according to the displayed peptide; some phage clones in the table have no peptide insert as indicated. The summary includes clones reported in Nguyen et al [41] as well as in this publication. Mutant clones were discovered as follows: a in Ph.D.-7 or Ph.D.-12 phage display experiments using Zn2+ as the target, b in serial amplification of the Ph.D.-7 library, c in a 135-minute screen of the serially amplified Ph.D.-7 or Ph.D.-12 library, d in the amplification of M13KE, e as a contaminating clone in a concurrent experiment in our lab. One column indicates clones that were also sequenced at position 5091: Y means that the T5091C mutation is present (also indicated by an asterisk * next to the name of the clone), N means that there is no T5091C mutation, and ND means that the clone was not sequenced at position 5091. The log(pfu/μL) at 135 minutes of incubation with E. coli cells are the same values plotted in the graphs of the respective publications (results corresponding to Fig 2B of the current work are underlined; results from Nguyen et al are not underlined; ND = not determined). Differences between old and new data for the same clone are due to small variations in conditions when experiments are performed on separate occasions, and they are generally not statistically significant. Each “x” indicates that the peptide has been identified in panning experiments reported in the literature and/or is present in the databases MimoDB, SAROTUP, and/or PhD7Faster, which is part of the SAROTUP suite (see details in the Discussion of Nguyen et al [41]). The last column contains the abundances of certain clones in the naïve library determined through deep sequencing (number of occurrences divided by 4 x 10^6^ clones analyzed) as reported by Derda and coworkers [76]. Clones without abundances w [file pone.0176421.s003.docx]

| **Name of Clone** | **Disc.** | **5’-UTR**  **Mutation** | **T5091C**  **?** | **5’-UTR of gene II mRNA** | **log(pfu/μL)**  **at 135-min** | **Lit.** | **Mimo-DB** | **SAROTUP**  **(PhDFaster)** | **Naïve**  **Abund.** |
| --- | --- | --- | --- | --- | --- | --- | --- | --- | --- |
| Normal M13KE Phage  Wild-type M13 |  |  | N  5092C | ***GUUUUUGGGGCUUUUC***UGAUUAUC**AACCGGGGUA**CAUAUG… | 5.38/5.26  7.51/7.59 |  |  |  |  |
| Ph-HAIYPRH  Ph-AKIDART  Ph-HTPQVHHPELTH  Ph-VTAHGGR | a, b  b  a  c | G6813A | N  ND  ND  ND | -----------------------------A----------… | 7.45  7.45  7.52  ND | x | x | x | 0.00053  0.00023  0.00016 |
| Ph-HQLHHHL  Ph-SDLVLRP | a  c | C6810T | ND  ND | --------------------------U-------------… | 7.18  ND |  |  | x |  |
| Ph-ANTLRSP  Ph-NoPeptide  Ph-HLHDTNH | b  c  a | A6809C | N  ND  ND | -------------------------C--------------… | 7.50  7.13  7.26 | x | x | (x) | 0.00004 |
| Ph-SNHAPRH* | c | A6802T | Y | ------------------U---------------------… | 6.91 |  |  |  |  |
| Ph-HEASQHAFSARL | c | C6799T | ND | ---------------U------------------------… | 6.71 |  |  |  |  |
| Ph-ARPPASP  Ph-QLHRHHH  Ph-HSHHHSA  Ph-SLLSHNS | c  a  a  e | T6798C | ND  N  ND  ND | --------------C-------------------------… | 7.01  6.68  7.36  6.59 | x | x | x  x (x) | 8x10^-7^ |
| Ph-HPPHHNT  Ph-AMSPRMDGKVFA  Ph-HLHRLHTHEHSK | a  c  a | T6798Δ | ND  N  ND | --------------Δ-------------------------… | 6.86  7.11  7.04 |  |  | (x)  (x) | 3x10^-6^ |
| Ph-TGITNWEVRTSR | a | C6794A | ND | ----------A-----------------------------… | 7.05 |  |  |  |  |
| Ph-LSMTGRD*  Ph-GWKSHLTHHHAE  Ph-NoPeptide | e  a  d | G6793A | Y  N  ND | ---------A------------------------------… | 7.19  6.84  7.32 |  |  | x |  |
| Ph-SRITIDN | c | G6793T | N | ---------U------------------------------… | 7.39 |  |  |  |  |
| Ph-HAFPHLH*  Ph-NoPeptide | a  d | G6793Δ | Y  N | ---------Δ------------------------------… | 6.95/7.23  7.33/6.68 | x |  |  |  |
| Ph-GKPMPPM  Ph-HDHRYPK  Ph-KLPGWSG*  Ph-KDTNIYDQRYSR | c  a  b  c | G6792T | N  ND  Y  ND | --------U-------------------------------… | 7.69  7.68  7.69  7.49 | x  x  x | x  x  x | (x)  (x) | 0.0014  0.00007  0.00008 |
| Ph-SPTQPKS* | c | G6792C | Y | --------C-------------------------------… | 7.19 |  |  |  | 0.00045 |
| Ph-HLFHLTPYHGHE | a | G6748Δ | N | Upstream of 5’-UTR (gene II promoter) | 7.15 |  |  | (x) |  |
| Ph-TLITFHHHEHVN | a | C6589T | N | Upstream of 5’-UTR (lacZα insertion) | 7.17 |  |  |  |  |
