# Supplementary material for: Various mutations compensate for a deleterious lacZα insert in the replication enhancer of M13 bacteriophage
Source: PLoS One. 2017 Apr 26;12(4):e0176421. doi: 10.1371/journal.pone.0176421 (PMC5405960; doi:10.1371/journal.pone.0176421)
Supplement: S1 Fig — One ΔlacZα-827 clone arose spontaneously from the ACX5X1 library and the other arose spontaneously from the ACX6C library, and are indicated as such. Each phage clone was amplified separately in an ER2738 culture. At 135 minutes, three aliquots from each flask of growing culture were diluted and plated, and the concentration of phage (pfu/μL) was determined based on plaque counts. The M13KE control was run 16 times for a total of n = 48 platings. WT-M13 was run 6 times (n = 18 platings), and all other phage clones were run twice each (n = 6 platings). Each bar represents the mean log(pfu/μL) of all platings for a given clone, and the error bars show the 95% confidence interval. Statistical analysis indicated significant differences among the phage concentrations for all the data sets (ANOVA; F5,89 = 212.3, P < 0.0001). Post-hoc analysis showed that the 135-min concentration for the ΔlacZα-827 ejection from the ACX5CX1 library is significantly higher than that of the library from which it arose (Tukey’s HSD; α = 0.05, P < 0.0001). Similarly, the ΔlacZα-827 ejection from the ACX6C library is significantly higher than its library (P < 0.0001). Both ejections are not statistically different from WT-M13 (P > 0.85). (PPTX) [file pone.0176421.s004.pptx]

## Slide 1
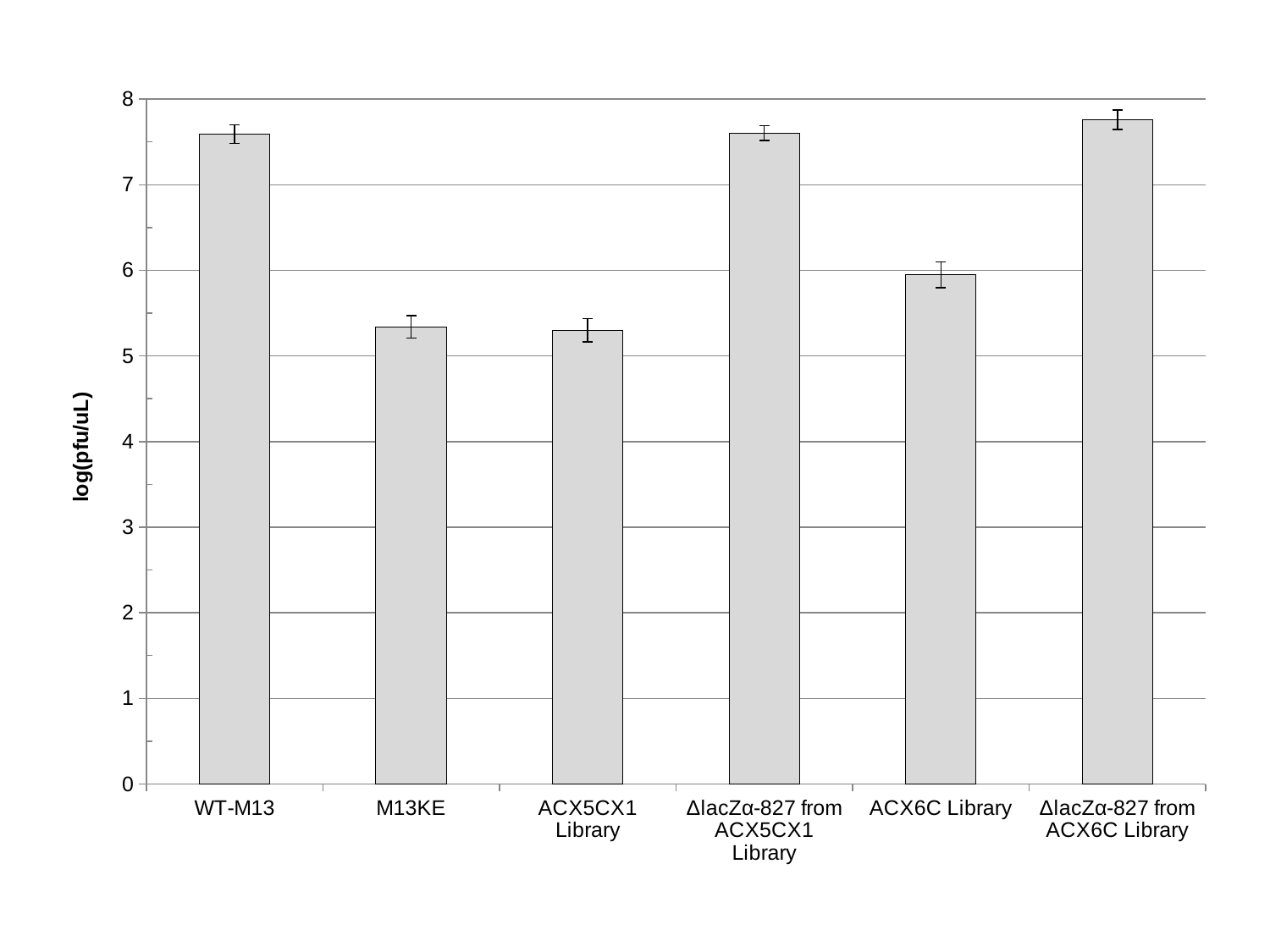

### Chart
| Category | |
|---|---|
| WT-M13 | 7.59164136338823 |
| M13KE | 5.337799263346661 |
| ACX5CX1 Library | 5.299675726090814 |
| ΔlacZα-827 from ACX5CX1 Library | 7.604203990622578 |
| ACX6C Library | 5.94916275164684 |
| ΔlacZα-827 from ACX6C Library | 7.760144118630762 |
