# Supplementary material for: Various mutations compensate for a deleterious lacZα insert in the replication enhancer of M13 bacteriophage
Source: PLoS One. 2017 Apr 26;12(4):e0176421. doi: 10.1371/journal.pone.0176421 (PMC5405960; doi:10.1371/journal.pone.0176421)
Supplement: S2 Fig — Sequences of the gene II 5’-UTR through the start codon were entered in the RNA folding form on the mfold web server [46]. All default parameters were used. In each case, the Structure 1 pdf was opened, and the image was copied into this document. Mutations and deletions are indicated by yellow circles and stars, respectively. The mutations shown have arisen spontaneously from the Ph.D.-7 and Ph.D.-12 libraries or from M13KE, with the exceptions of U6797C and U6797C+U6789Δ. We attempted to synthetically incorporate U6797C into M13KE based on its similar secondary structure to G6792U. We instead obtained U6797C+U6789Δ, which has a predicted secondary structure that is essentially the same as that predicted for U6797C. ΔGfold is the free energy change for folding. (PPTX) [file pone.0176421.s005.pptx]

## Slide 1
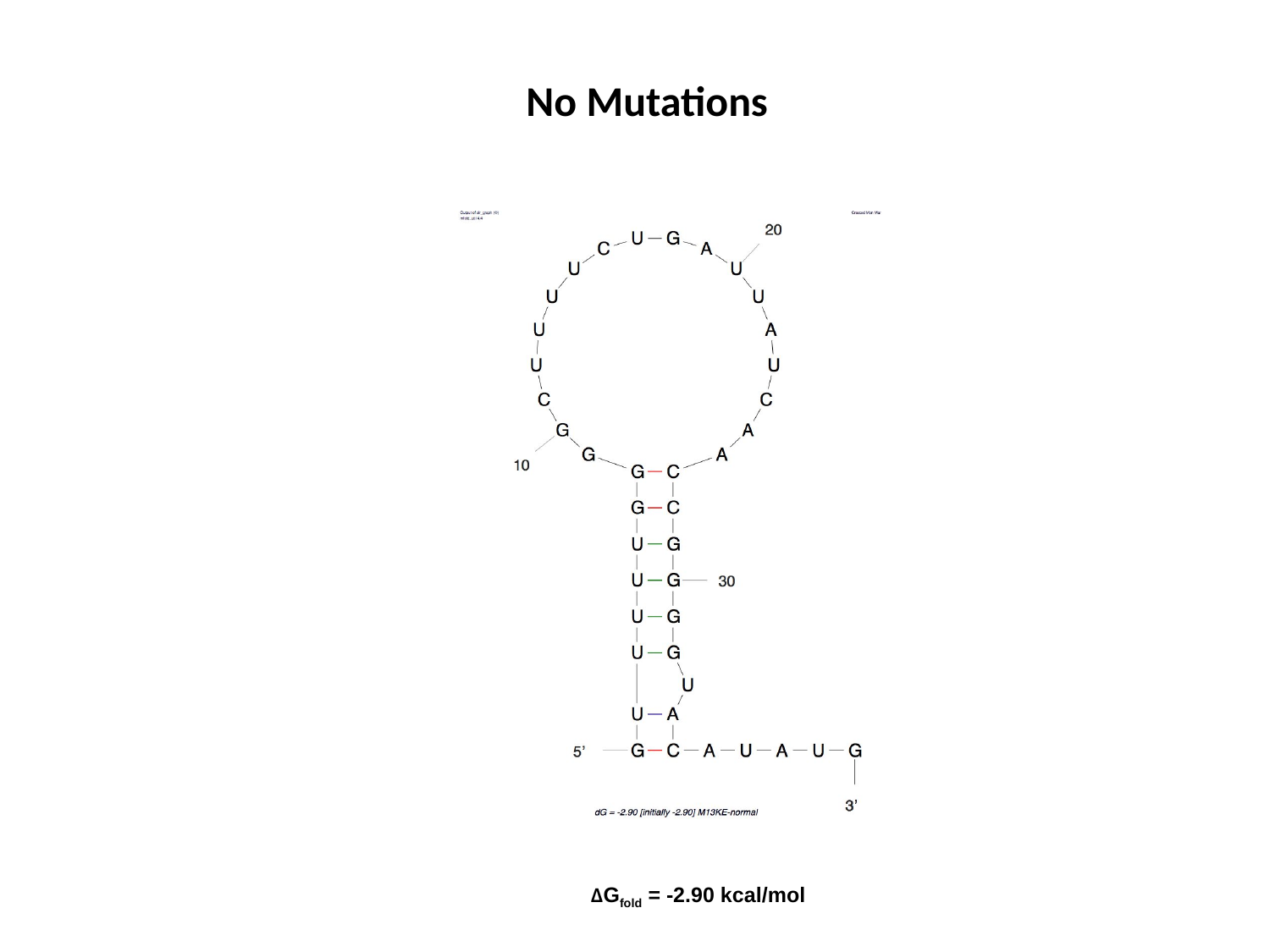

No Mutations
ΔGfold = -2.90 kcal/mol

## Slide 2
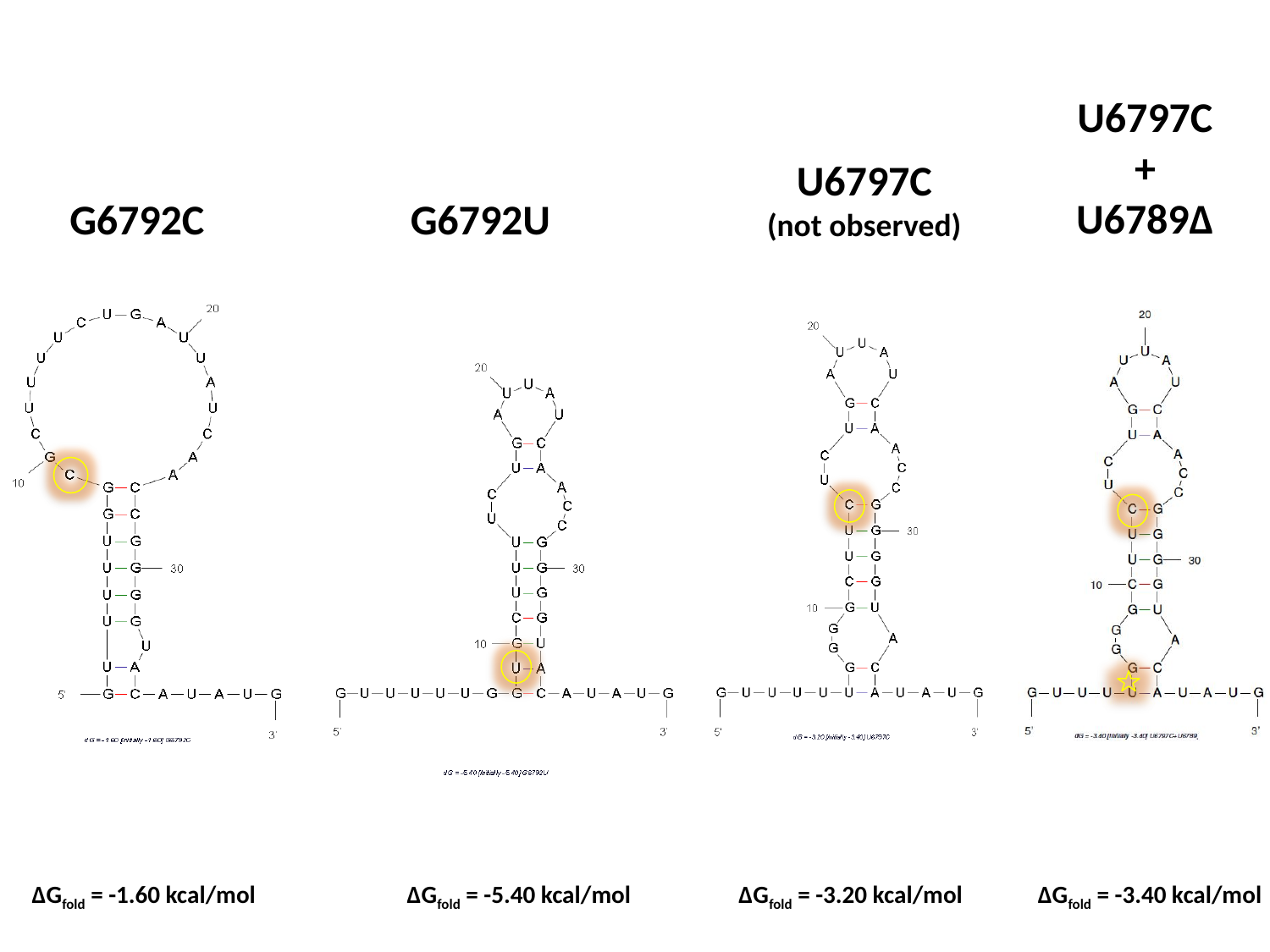

U6797C
+
U6789Δ
U6797C
(not observed)
G6792C
G6792U
ΔGfold = -1.60 kcal/mol
ΔGfold = -5.40 kcal/mol
ΔGfold = -3.20 kcal/mol
ΔGfold = -3.40 kcal/mol

## Slide 3
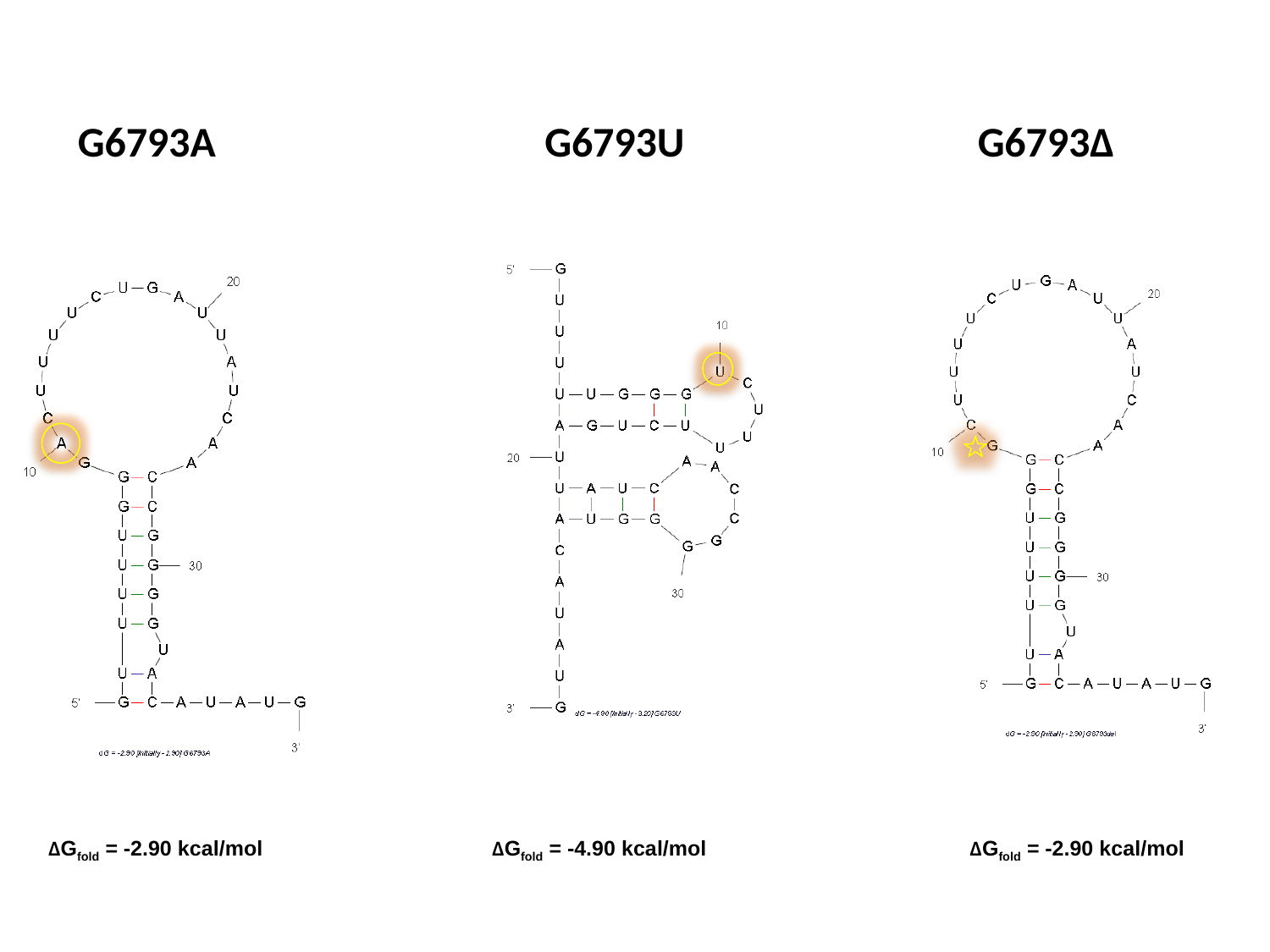

G6793A
G6793U
G6793Δ
ΔGfold = -2.90 kcal/mol
ΔGfold = -4.90 kcal/mol
ΔGfold = -2.90 kcal/mol

## Slide 4
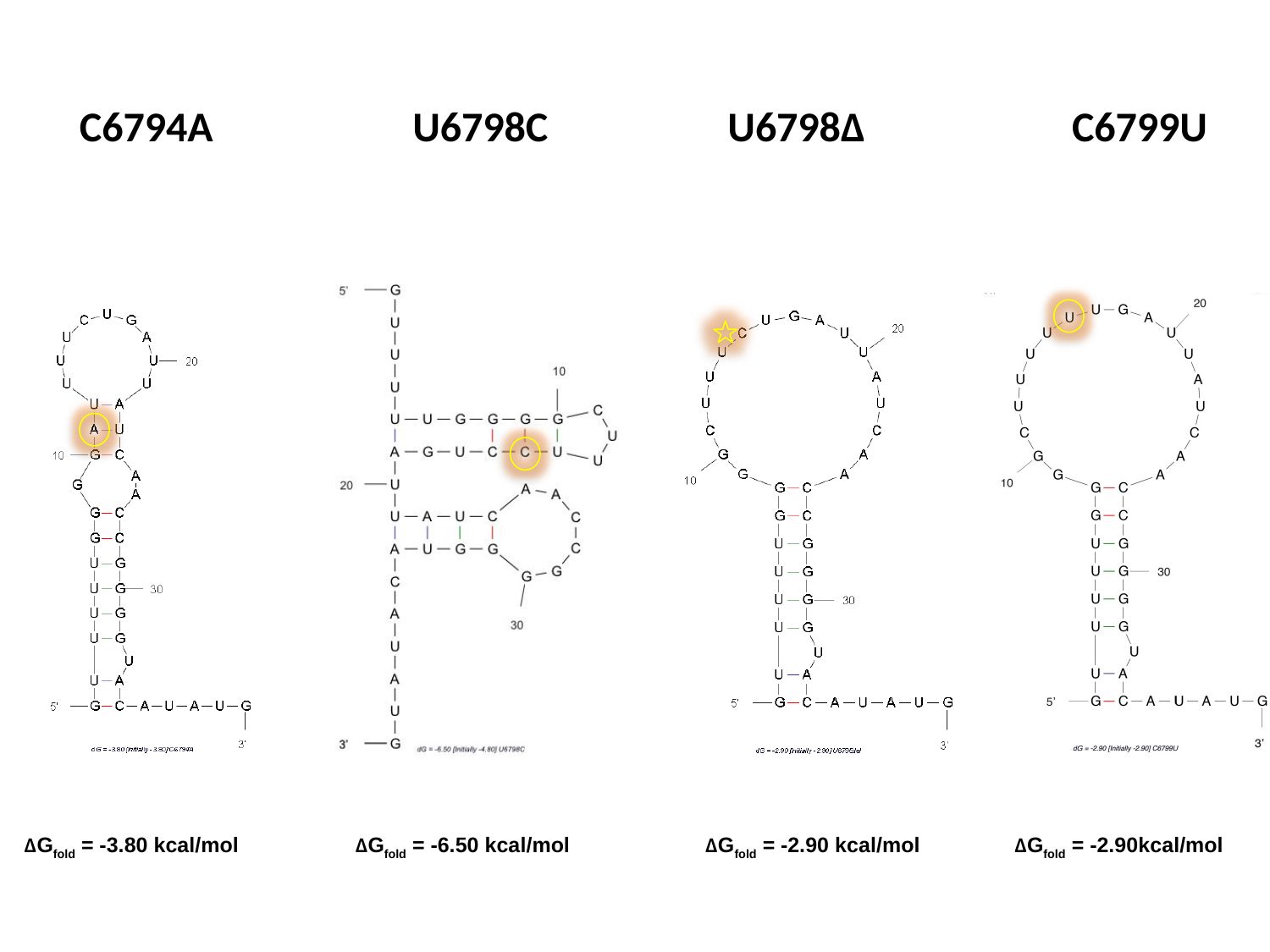

C6794A
U6798C
U6798Δ
C6799U
ΔGfold = -3.80 kcal/mol
ΔGfold = -6.50 kcal/mol
ΔGfold = -2.90 kcal/mol
ΔGfold = -2.90kcal/mol

## Slide 5
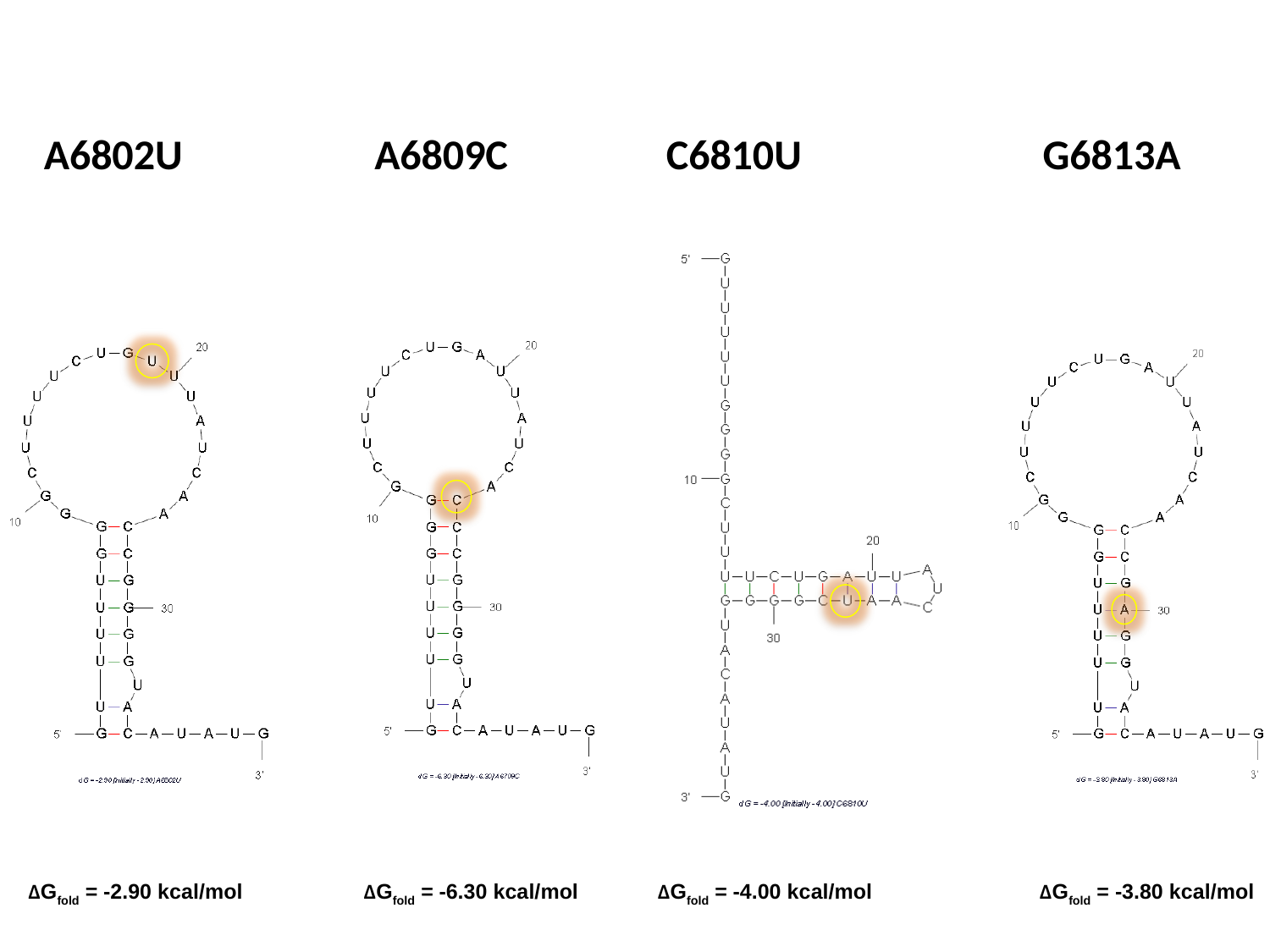

A6802U
A6809C
C6810U
G6813A
ΔGfold = -2.90 kcal/mol
ΔGfold = -6.30 kcal/mol
ΔGfold = -4.00 kcal/mol
ΔGfold = -3.80 kcal/mol
